# Diabetes and traditional remedies in Medieval Persia: a historical perspective

**DOI:** 10.3389/fphar.2025.1673440

**Published:** 2025-11-12

**Authors:** Fereshteh Safavi, Adolfo Andrade-Cetto, Sonia M. Escandón-Rivera

**Affiliations:** Laboratorio de Etnofarmacología, Departamento de Biología Celular, Facultad de Ciencias, Universidad Nacional Autónoma de México, Ciudad de México, Mexico

**Keywords:** traditional Persian medicine, diabetes, temperament, hot diabetes, cold diabetes

## Abstract

Traditional Persian Medicine (TPM) has long contributed to the diagnosis, prevention, and treatment of diabetes. Rooted in the theory of temperament, TPM classifies individuals and diseases according to four qualities: warm-dry, warm-wet, cold-wet, and cold-dry. In classical TPM literature, diabetes is referred to by various names—most notably “dhiabitos” and “dulāb”—and is consistently associated with dysfunction of the kidneys and liver. For this study, seminal TPM texts were systematically reviewed using targeted keywords to extract definitions, etiologies, symptoms, diagnostic criteria, and therapeutic strategies related to diabetes. These findings were supplemented with data from electronic databases, including ScienceDirect, PubMed, and Scopus. TPM classifies diabetes into two categories: hot and cold. Historical manuscripts detail a wide array of therapeutic preparations, predominantly herbal and herbo-mineral formulations, along with several mineral substances believed to balance temperament. Among these, oral dosage forms, particularly Safoofs (dry powders), were the most frequently prescribed across both categories. In total, 208 plant species from 81 botanical families, along with several mineral substances, were identified in traditional formulations. According to TPM, the medicinal plants, similarly to diseases, commonly exhibit either hot or cold properties, reflecting the principle that treatments should rebalance underlying dystemperament rather than merely relieve symptoms. This paradigm emphasizes addressing the root causes of disease. TPM physicians (hakims) often attribute diabetes to factors such as kidney dysfunction and dysregulated water metabolism. In contrast, modern biomedical literature does not distinguish between “hot” and “cold” diabetes; these classifications may reflect two stages in the spectrum of diabetes pathology. Hot diabetes may align with the early stages of Type 2 diabetes, while cold diabetes corresponds to the advanced stages of Type 2 diabetes or possibly Type 1 diabetes. Future research should examine the phytochemical and pharmacological profiles of plants used in the treatment of “hot” and “cold” diabetes, with a particular emphasis on their hypoglycemic properties. Plants used for “hot” diabetes may possess anti-inflammatory effects that protect pancreatic beta cells and improve insulin function. Additionally, it is important to explore the potential effectiveness of plants associated with “cold” diabetes in managing diabetes-related complications, such as nephropathy.

## 1 Introduction

### 1.1 Definition and overview of diabetes: a modern perspective

Globally, approximately 589 million adults aged 20–79 are living with diabetes mellitus. Elevated blood glucose levels occur when the body does not produce enough insulin, cannot effectively utilize the insulin produced, or both. Type 1 diabetes is typically an autoimmune condition characterized by the destruction of insulin-producing β-cells in the pancreas, resulting in an absolute deficiency of insulin. Although the exact causes remain unclear, both genetic predisposition and environmental factors play major roles. Thanks to modern treatments, individuals with type 1 diabetes can lead healthier lives and significantly reduce their risk of complications (ADA, 2025; IDF, 2025). Type 2 diabetes accounts for approximately 90% of all diabetes cases worldwide and is projected to become the second leading cause of disease burden by 2050. It is characterized by insulin resistance, a condition in which the body’s cells fail to respond effectively to insulin, prompting the pancreas to produce increasing amounts of insulin to maintain euglycemia. Over time, β-cell function may decline due to chronic overstimulation, leading to progressive insulin deficiency. Key risk factors include obesity, aging, ethnicity, and family history. Management primarily emphasizes lifestyle modifications, such as adopting a nutritious diet, engaging in regular physical activity, smoking cessation, and maintaining a healthy weight. When lifestyle interventions prove insufficient, oral agents such as metformin are commonly prescribed as first-line therapy. If target glucose levels remain unmet, dual therapy may be considered to enhance glycemic control, including sulfonylureas, thiazolidinediones, dipeptidyl peptidase-4 (DPP-4) inhibitors, glucagon-like peptide-1 (GLP-1) receptor agonists, glucose-dependent insulinotropic polypeptide (GIP), and sodium-glucose co-transporter 2 (SGLT2) inhibitors (IDF, 2025).

Herbal medicine has been a significant and widespread facet of complementary and alternative medicine for thousands of years. Diabetics often turn to these medicines, either as a complementary therapy or as the main approach to improving their condition (Cruz and Andrade-Cetto, 2015; Itrat and Akhlaq, 2022). In this context, the rich reservoir of traditional medical manuscripts and local healers’ practices offer invaluable insights (Shankar and Liao, 2004). TPM, also known as “Sinai medicine,” represents a holistic medicine tradition whose history spans several centuries (Dabaghian et al., 2012). The World Health Organization acknowledges the significance of traditional medicines and supports their incorporation into national healthcare systems (WHO, 2023). Iran holds the third position in traditional medicine trials on the International Clinical Trials Registry Platform (ICTRP), highlighting its pioneering status in this field, following China and India (Juneja et al., 2020). In the annals of medical history, diabetes mellitus unfolds a fascinating story dating back 3,500 years. Diabetes diagnosis and treatment were extensively studied in Persian literature and clinical settings during the Middle Ages (Larijani et al., 2016). Sections 1.2 and 1.3 provide a concise overview of “TPM” and “Temperament,” underscoring their relevance in framing the present study. This review offers a comprehensive examination of diabetes through the lens of TPM, addressing its definition, cultural context, and the significance of temperament theory in disease interpretation. A central aim is to identify and describe, in detail, the historically utilized dosage forms and constituents of herbal preparations. Regardless of current evidence regarding efficacy, this study explores the properties, nature, and temperament of their ingredients. Herbal, mineral, and animal-derived components found in traditional formulations were systematically extracted, with rigorous efforts to match each historically named plant to its modern common or scientific nomenclature. By revisiting the historical application of TPM in diabetes management, we aim to encourage contemporary practitioners to draw insights from this ancient corpus of knowledge. Furthermore, we hope to foster deeper inquiry into conventional therapeutic approaches. To our knowledge, this is the first review to integrate these specific objectives, underscoring our unique contribution and opening new avenues for future research.

### 1.2 A brief historical background to TPM

As a holistic medical system, TPM has a long and fruitful history, originating in ancient Iran. Since ancient times, TPM has played a significant role in the diagnosis, prevention, and treatment of diseases in Persia (Rezaeizadeh et al., 2009). Rooted in practical experience and observations passed down through generations, TPM has evolved over time and has been enriched by medical knowledge from civilizations such as India, China, Egypt, and Greece (Gorji and Khaleghi Ghadiri, 2001). The TPM healers, known as “Hakims,” contributed to the development of the theory of the four humors, known as “Ákhlāt” in Persian, which later became a fundamental concept in global medicine (see section 1.3 for details) (Zargaran, 2014). Maintaining a balance between each humor has been crucial for body health. In TPM, the six basic principles (Sette-ye-Zarurieyeh) are considered the most significant factors in maintaining or losing health, which together form major aspects of a lifestyle. These factors include weather, food and drink, exercise, retention and excretion, sleep and wakefulness, and mental and emotional state (Aghili Alavi Khorasani Shirazi, 2006; Iranzadasl et al., 2021). According to this ancient doctrine, “medicine” refers to the knowledge of maintaining health and treating illness, with the former taking precedence (Arzani, 1915). In this respect, the TPM manuscripts, mostly written by Persian scholars in the Middle Ages, offer a treasure trove of knowledge about traditional medicine and pharmaceuticals, as well as an array of inventive studies, particularly about using medicinal plants in medicine (Schulz, 2023). For instance, the Qarabadin books are among the best-known pharmaceutical texts written by Persian scholars. These traditional pharmacopeias contain meticulously comprehensive lists of herbal medicine names, formulas and recipes describing how to prepare compound medicines. Qarabadin formulas differ from author to author.

### 1.3 Temperament theory in TPM: disease etiology

In TPM, fire, air, water, and earth are considered the four fundamental elements present in both human and non-human entities. Warmth, coldness, wetness, and dryness are the basic qualities associated with each of the elements. Fire is characterized by warmth and dryness, air by warmth and wetness, water by coldness and wetness, and earth by coldness and dryness. When these elements interact within composite bodies, a dominant configuration of qualities—referred to as “temperament” (Mezaj)—emerges, shaping the physiological and pathological nature of the organism (Avicenna, 2005). Temperament comprises four humors (khelts): “warm-dry (Sáfrā or yellow bile),” “warm-wet (Dám or blood),” “cold-wet (Bálghám or phlegm),” and “cold-dry (Sáudā or black bile)” (Naseri et al., 2009). Consequently, various substances—including medicines, herbs, and foods—possess distinct temperaments derived from the elemental qualities of heat, cold, wetness, and dryness (Avicenna, 2005). These temperaments influence physiological functions and pathological conditions in the human body. Within the framework of TPM, diseases are diagnosed based on an individual’s inherent temperament. An imbalance among these qualities is believed to precipitate disease, and treatment strategies aim to restore equilibrium through targeted dietary, herbal, and lifestyle interventions (Naseri et al., 2009).

### 1.4 Treatment approaches to diabetes in TPM

#### 1.4.1 The importance of medicinal plants in diabetes treatment in TPM

Herbal medicine encompasses a range of products, including raw herbs, herbal materials, herbal preparations, and finished herbal formulations. The last two categories, herbal preparations and finished herbal formulations, include herbal drugs and their preparations that serve as the active pharmaceutical ingredients (APIs). These substances are derived directly from botanical sources. “Mixture herbal products” refer to formulations containing more than one herb and may also include excipients to support stability or delivery. In certain regulatory frameworks, herbal medicines may incorporate naturally occurring organic or inorganic substances of non-plant origin such as those derived from animals or minerals (WHO, 2007). In TPM, herbal remedies are employed to treat both types of diabetes using either single-herb formulations or mixture herbal products. Each herb is attributed to a distinct temperament based on its elemental qualities; therefore, prescribing the same herbal medicine for different types of diabetes without first evaluating the patient’s temperament is considered ineffective and potentially harmful. Mixture herbal products are formulated to maintain a deliberate balance among the constituent ingredients, considering their individual temperaments. To achieve this, TPM developed principles for determining the overall temperament of compound preparations—particularly those containing more than ten botanicals with varying profiles. The four fundamental qualities—heat, cold, wetness, and dryness—are graded on a scale from 1 to 4, reflecting increasing potency. Hakims calculated the final temperament of a formulation by weighing the dose and grading of each herb, ensuring therapeutic equilibrium within the preparation (Kabir, 2003).

#### 1.4.2 Dosage form and mode of administration of herbal medicine

TPM manuscripts cover a wide range of therapeutic dosage forms and treatments. Diabetes dosage forms include several ones. These dosage forms are divided into two categories: oral and non-oral forms, each further subdivided.

## 2 Methodological approach

**FIGURE 1:**
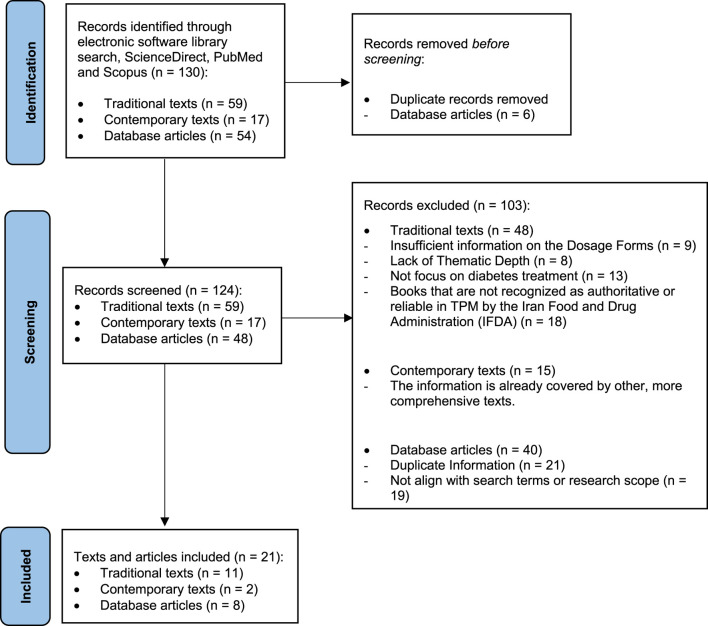
PRISMA flow diagram for included studies across three phases: historical text mining, botanical identification, and contemporary literature review.

This review was conducted in three distinct phases. The first phase, historical text mining, involved a targeted search of authoritative sources among surviving Persian manuscripts dating from the 9th to the 18th centuries. Traditional knowledge was systematically extracted using Noor software (version 1.5), a searchable digital library comprising approximately one thousand TPM texts. Sources exhibiting low thematic relevance or superficial similarity were excluded using a modified PRISMA methodology (Figure 1). Following this process, 11 traditional texts were selected based on rigorous screening and final evaluation of thematic relevance. These books, endorsed by the Iran Food and Drug Administration, are supported by empirical evidence, possess historical validity, and are grounded in the philosophical principles that form the foundation of TPM. These books include Al-Hawi (1) (Razi, 2005), Al-Qanun fi al-tibb (2) (Avicenna, 2005), Al-Aghraz Al-Tabiyeh (3) (Jorjani, 2010), Tohfato Al'-Momenin (4) (Tonekaboni, 2007), Qarabadin-e-Kabir (5) (Aghili Alavi Khorasani Shirazi, 2007), Makhzan-Al’ Advieh (6) (Aghili Alavi Khorasani Shirazi, 2008), Makhazen al Taalim (7) (Khan, 2015), Kamil al-Sana’ah al-Tibbiyya (8) (Ahwazi, 2009), Exir-e-Azam (9) (Chashti and Nazem, 2008), Qarabadin-e-Aazam (10) (Chashti and Nazem, 2004), and Tebe-Akbari (11) (Arzani, 2009). The review focused on identifying content related to diabetes by using search terms such as “Dhiabitos,” “Dulāb,” and other relevant names listed in Section 3.1. Relevant material was systematically extracted, including definitions of diabetes according to conventional medical frameworks, clinical signs and symptoms, the two types of diabetes as classified in TPM, along with their distinguishing features and corresponding treatments, dosage forms previously prescribed for each type, and principles guiding the formulation of compound medicines, along with supplementary information. Additionally, all herbal and mineral constituents referenced in these dosage forms were documented. For each medicinal plant cited, data were recorded on traditional name(s), plant part(s) used, and temperament classification.

In the second phase, botanical identification, significant effort was devoted to accurately assigning vernacular names to modern botanical taxa. Plant species cited in historical texts, mostly under Persian, Arabic, and Indian traditional names, were systematically compared with contemporary scientific classifications. To ensure the highest level of accuracy, the study relied on historical ethnobotanical identification literature, including several authoritative Persian treatises and ethnobotanical glossaries, to correlate ancient nomenclature with accepted botanical equivalents (Figure 1) (Tonekaboni, 2007; Aghili Alavi Khorasani Shirazi, 2008; Unani Pharmacopoeia of India, 2008; Ghahreman and Okhovvat, 2009).

The third phase, contemporary literature review, involved searches in modern scientific databases to supplement and contextualize findings. Regional flora databases [World Flora Online (www.worldfloraonline.org), International Plant Names Index (IPNI) (www.ipni.org), and the Medicinal Plant Names Services (MPNS)] were used to validate the individual identifications and to determine the family genera of each plant. In cases where the correspondence between the traditional name of a plant and its scientific name was uncertain and where several plants were referred to by that traditional name in contemporary texts, all relevant plants were mentioned. Scientific databases such as ScienceDirect, PubMed, and Scopus were also used for targeted searches for complex terms and to supplement conventional literature.

## 3 Results and discussion

### 3.1 Diabetes and herbal therapeutics in TPM

#### 3.1.1 Types of diabetes: TPM insights

“Dhiabitos,” a Greek word that entered Persian manuscripts, and “Dulāb,” a Persian word, describe the same symptoms of diabetes in the TPM books. Polyuria, polydipsia, and thirst are main symptoms. Dulāb in Persian means “water wheel” in which water is constantly turning from one side to the other, so diabetes was conceptualized as dulāb because of the constant thirst and polyuria (Arzani, 2009). Diabetes was introduced in TPM texts under other names, including “Diyanitas,” “Diasquoms,” “Dhiaskous,” and “Qaramis,” which are Greek words, and “Aldawaarat,” “Alduwlab,” “Zalaq al-kuliyyah,” “Zalaq al-Am’aa al-kuliyyah,” “Zalaq al-mizaj,” and “almaebar,” which are Arabic (Baghdadi, 1983). Considering the definition, dhiabitos was found to be strongly correlated with kidney and liver function. Apparently, the retention force of the kidneys (conceptualized as the kidney’s ability to concentrate urine) is reduced and may therefore be characterized by excessive urine output. The hot temperament of the kidneys led to an increase in the absorption of moisture and water from the liver and stomach into the kidneys. This phenomenon was found to be another etiologic symptom (Rezaeizadeh et al., 2009; Jorjani, 2010; Khan, 2015). In TPM, diabetes is divided into two categories: “hot diabetes” (Dhiabitos-e-har) and “cold diabetes” (Dhiabitos-e-bared), both with specific symptoms. From this perspective, diabetes patients have either hot or cold dystemperaments (Sou-e-mezaj), so diagnosis and treatment are based on clinical symptoms. Table 1 lists the symptoms of these two diabetes types. In the past, hot diabetes had the highest number of diabetics. In TPM, herbs that cleanse and hydrate the liver and reduce excessive heat play a crucial role in the treatment of hot diabetes. These herbs are believed to help remove excess bile from the blood, balance the liver’s temperament, enhance the kidneys’ ability to eliminate excess heat and toxins from the body, and reduce symptoms associated with hot diabetes (Chashti and Nazem, 2004; 2008). The TPM text states that cold diabetes is caused by Sáudā dominance in the body and is more common in women and obese people. It is said that it occurs under stress (Avicenna, 2005; Chashti and Nazem, 2008; Jorjani, 2010). As part of TPM treatment, strengthening the heart, brain, and liver and eliminating Sáudā side effects are among the measures used to treat cold diabetes.

**TABLE 1:** TPM classification of diabetes.

Type	Symptoms
Hot diabetes	Severe thirst, frequent urination and incontinence, colored urine without dysuria, a rapid pulse, excessive increase in libido, weight loss or emaciation, and a feeling of warmth in the back (in the kidney area).
Cold diabetes	Thirst (but less than in hot diabetes), pale urine without dysuria, coldness in the kidney area or throughout the body, weakness, loss or decrease in libido, signs of weight loss, decreased energy, and appetite, kidney disorders, a slow pulse, constipation, indigestion, reflux, and tingling in the feet and hands.

#### 3.1.2 Bridging traditional and modern diabetes perspectives through symptomatology

The classification of diseases varies across cultures, reflecting differing conceptual perceptions of illness. In traditional medicine, diagnosis is generally symptom-based, whereas modern medicine relies on an integrative approach that includes clinical symptoms, laboratory analyses, imaging techniques, and functional assessments. In this context, our understanding of diabetes and its treatment has evolved remarkably over the last century. From the identification of the pancreas’s role in the 19th century to the recognition of insulin resistance as a key factor in type 2 diabetes during the 1980s, our conceptual framework has undergone a profound transformation (March et al., 2022). According to the most important manuscripts in TPM, the disease dhiabitos and what is now known as diabetes mellitus share some similarities. Weight loss, unusual thirst, and polyuria were some of the main similarities. However, modern literature generally does not differentiate between hot and cold diabetes, so the term “hot and cold diabetes” may not actually refer to two distinct types of diabetes, such as Type 1 diabetes or Type 2 diabetes, but rather to two stages on the spectrum of diabetes pathology. The symptomatology described in Table 1 shows that at least three main symptoms, such as frequent urination, intense thirst, and weight loss, can reflect the early stages of Type 2 diabetes, suggesting that hakims’ concept of hot diabetes may be relevant in this regard. A fast heart rate (tachycardia) is not exclusively associated with diabetes but can be alarming when present with polyuria and other diabetes symptoms. These symptoms are common in people with diabetes mellitus with poorly controlled blood glucose levels. High blood glucose levels can lead to various complications, including cardiovascular and kidney problems. In an attempt to excrete excess glucose, it can lead to dehydration and trigger a rapid heart rate (Harkins, 2016). In contrast, the symptoms of cold diabetes such as moderate thirst, sexual dysfunction, neuropathy-related symptoms such as tingling, and gastrointestinal problems such as constipation, indigestion, and reflux can indicate either the advanced stage of Type 2 diabetes or possibly Type 1 diabetes (ADA, 2025; ElSayed et al., 2025). This distinction may provide insight into the increased libido observed in individuals with hot diabetes. Since hot diabetes is associated with an elevated metabolic state, early-stage patients may experience heightened energy levels, potentially leading to an increase in libido. Comparing libido between individuals with hot diabetes and those with cold diabetes, it is likely that the former exhibit higher levels of sexual desire. Therefore, it is conceivable that hakims observed and documented libido levels in relation to the distinct conditions of hot and cold diabetes rather than against a baseline of normal physiological function in healthy individuals. To refine our understanding of traditional diabetes concepts, it is essential to interpret the pharmacological and phytochemical properties of the plants used in the past to treat these diseases. It is likely that such an analysis will give us a better understanding of how these two types of diabetes in TPM fit into the modern and advanced definition of diabetes, provide deeper insights into the mechanisms of action and therapeutic effects of these drugs, and bridge the gap between traditional knowledge and modern science.

#### 3.1.3 Relationship between the plants recommended in TPM for diabetes treatment and their therapeutic properties

This correlation highlights the multifaceted rationale underlying plant selection in TPM for diabetes management. Several therapeutic qualities recur among the medicinal plants traditionally recommended, each contributing to symptom relief or modulation of disease mechanisms. Astringent plants are commonly prescribed to counteract renal tubule dilation by promoting contraction, thereby aiding water retention. The laxative plants counter the average patient’s tendency toward dryness, which can lead to constipation. Therefore, these plants do not address the disease itself, but rather one of its symptoms. Strengthening plants help alleviate kidney weakness by enhancing renal tubule tone and preventing excess water loss, supporting the kidneys’ overall strength and functionality. Cold and cooling plants are indicated to mitigate excessive renal heat, which draws in water, while hot and warming plants target general bodily or hepatic/renal coldness, believed to impair kidney function. Moisturizing plants, in turn, alleviate persistent thirst, one of the hallmark symptoms in diabetic conditions (Paavilainen, 2009). Table 2 presents a summary of these traditional therapeutic categories along with representative plant species associated with each quality. These therapeutic properties are aimed at combating both the causes and symptoms of diabetes. They are designed to treat diabetes caused by an imbalance of cold and heat, although the treatment of cold-induced diabetes obviously takes precedence (Paavilainen, 2009). Different medieval healers held different views on diabetes treatment therapeutic qualities, resulting in various treatment formulas. A comparison of their theories reveals two universally recommended medicinal qualities: “cold” and “hot.” These qualities aim to counteract the main causes of diabetes, heat and cold, and emphasize the importance of addressing the causes of the disease and not just treating the symptoms. In the TPM, each medicinal plant mentioned has at least one of these qualities. *Boswellia sacra* Flück., for example, has three qualities, namely, astringent, hot and warming, and strengthening or *Aloe vera* (L.) Burm. f. (Warm-dry) has two qualities: astringent and laxative. Interestingly, the number of qualities associated with a plant has no significant impact on its popularity if it has at least one suitable quality. In this regard, diabetes therapy primarily targets what it perceives as external causes of kidney weakness and water retention, namely, heat and cold. In cold diabetes, where the weakening of the kidney and its ability to retain moisture are impaired, TPM recommends the use of plants with a hot or warming temperament to counteract this effect. In addition, rubefacient plants or alternatively astringent plants are recommended to constrict the kidney channels and support moisture retention. Strengthening plants are also recommended to address kidney weakness in these cases (Avicenna, 2005).

**TABLE 2:** Therapeutic qualities of plants recommended in TPM for diabetes treatment.

Therapeutic quality	Plants examples
Astringent	*Vachellia nilotica* subsp. tomentosa (Benth.) Kyal. & Boatwr., *Prunus amygdalus* Batsch, *Aloe vera* (L.) Burm. f., *Malus domestica* (Suckow) Borkh, *Plantago indica* L., *Boswellia sacra* Flück., *Quercus infectoria* G. Olivier, *Vitis vinifera* L., *Polygonum aviculare* L., *Cistus ladanifer* L., *Crataegus germanica* (L.) Kuntze, *Mentha piperita* L., Morus spp., *Myrtus communis* L., *Prunus domestica* L., *Punica granatum* L., *Cydonia oblonga* Mill., *Rosa × damascena* Herrm., *Tragopogon porrifolius* L., and *Bambusa bambos* (L.) Voss
Laxative	*Aloe vera* (L.) Burm. f., *Plantago indica* L., *Lactuca sativa* L., Morus spp., *Myrtus communis* L., *Prunus domestica* L., *Punica granatum* L., *Papaver somniferum* L., *Cucurbita pepo* L., *Cydonia oblonga* Mill., *Raphanus raphanistrum* subsp. sativus (L.) Domin, *Rosa × damascena* Herrm., *Nymphaea alba* L., *Prunus amygdalus* Batsch, *Althaea officinalis* L., *Cucumis sativus* L., and *Foeniculum vulgare* Mill
Strengthening	*Malus domestica* (Suckow) Borkh, *Boswellia sacra* Flück, Mentha piperita L., *Myrtus communis* L., *Punica granatum* L., *Cydonia oblonga* Mill., *Rosa × damascena* Herrm., *Tragopogon porrifolius* L., *Bambusa bambos* (L.) Voss, *Aloe vera* (L.) Burm. f., *Mangifera indica* L., and *Cinnamomum burmanni* (Nees & T. Nees) Blume
Cold and cooling	*Vachellia nilotica* subsp. tomentosa (Benth.) Kyal. & Boatwr., *Hordeum vulgare* L., *Quercus infectoria* G. Olivier, *Polygonum aviculare* L., *Lactuca sativa* L., Morus spp., *Myrtus communis* L., *Prunus domestica* L., *Papaver somniferum* L., and *Bambusa bambos* (L.) Voss
Hot and warming	*Prunus amygdalus* Batsch, *Boswellia sacra* Flück, *Vitis vinifera* L., *Cistus ladanifer* L., *Mentha piperita* L., *Myrtus communis* L., *Punica granatum* L., and *Mentha piperita* L. Rarefying plants such as *Vachellia nilotica* subsp. tomentosa (Benth.) Kyal. & Boatwr., *Prunus amygdalus* Batsch, *Prunus domestica* L., *Raphanus raphanistrum* subsp. sativus (L.) Domin, and *Nymphaea alba* L.
Moisturizing	*Malus domestica* (Suckow) Borkh, *Hordeum vulgare* L., *Mentha piperita* L., Morus spp., *Prunus domestica* L., and *Cydonia oblonga* Mill.

### 3.2 Dosage form and mode of administration of medicines at TPM

Persian scholars have named many natural pharmaceutical dosage forms from the remaining Persian books. They are determined not only by the pharmaceutical form, but also by the route of administration, ingredients, and therapeutic effect. Over 226 dosage forms are mentioned in the TPM literature. The final list of dosage forms was reduced to nearly 60 items, as many were related only to the preparation methods (Baranifard et al., 2017). Table 3 summarizes the most common oral and non-oral dosage forms used to treat hot and cold diabetes in TPM, based on data from the texts included in this paper. It contains the names and definitions of the herbal mixture products selected from the reviewed traditional texts. It seems that in that time, oral dosage forms, especially safoofs, dried oral powders, were the most prescribed dosage forms for diabetes and kidney disease. Table 4 lists the ingredients in each mixture herbal product, as well as the amount of each herb required for the formulation. Additionally, preparation methods are summarized. Despite their standard names, weights and measures were used differently during different historical periods, communities, and regions. Furthermore, pharmacological weights can be classified in three groups: specific, semi-specific, or non-specific. A specific weight can be converted to the metric system. Weights are defined as semi-specific if they refer to a cupful, a few handfuls, or the weight of a nut or lentil, or as non-specific when referring to terms such as “part” (juz’i), “quantity” (meqdār), and the like (Kahl, 2007). Non-specific weights enabled practitioners to adjust formulations according to the resources at hand or the situation, which is essential in traditional practices where precise measurements were often impractical. These terms illustrate a cultural perspective on measurement that contrasts with contemporary standards. All the prescriptions studied in Table 4 used specific weights, except in a few cases where semi-specific weights were used. Table 5 gives conversion factors for each traditional weight unit to its modern equivalent (Fakhouri and Khowam, 2018). These findings reveal the medieval history of TPM pharmacy.

**TABLE 3:** Common dosage forms for diabetes in TPM.

Rout of administration		Traditional dosage forms	Definition	Mixture herbal products name	
				Cold diabetes	Hot diabetes
Oral	Liquids	Sharbat/ sharāb	Herbal syrups or tonics	Sharbat-e-bozouri har 1 Sharbat-e-bozouri har 2 Ma’al osool	Sharbat-e-onnab Sharbat-e-khashkhash Sharbat-e-Muarreh
	Semi-Solids/ viscose liquids	Jawaresh	Herbal formulations prepared for food digestion	—	Javaresh-e-ameleh
		Majoon	Semi-solid pastes solidified by honey or similar substance	Methroditus Majoon-e-falaafeli	Mask albawl khamireye abrisham
	Solids	Safoof	A dry mixture of triturated herbal medicines	Mask-albawl-e-Har Safoof-e-zaranb	Safoof dafe’e dhiabitos 1 Safoof dafe’e dhiabitos 2 Safoof dafe’e dhiabitos 3 Safoof dafe’e dhiabitos 4 Safoof dafe’e dhiabitos 5 Safoof-e− qali Safoof-e-Galoo Safoof by Hakim Waris Ali Khan
		Qors	Tablets. In their preparation, gums such as tragacanth and Arabic gum are commonly used	Dhiabitos 1 Dhiabitos 2 Dhiabitos 3 Tabaashir	Qors-e-Golnar Qors-e-Kafour Nafs-ol dam Boul-ol dam
Non-Oral	Transdermal	Zemad	Ointment-like medicines prepared as mushy liquids	—	Zemad-e-sandal Zemad for dhiabitos
		Abzan	Sitz bath, Immersion bath; An abzan is a metal, wooden or earthen vessel the size of a person with a perforated lid in which the patient’s head protrudes from the hole.	—	Abzan nafe’e dhiabitos

**TABLE 4:** Ingredients and preparation methods for mixture herbal products used in diabetes treatment in TPM.

Cold diabetes	Herbal formulation ingredients	Preparation
Sharbat-e-bozouri har 1	*Foeniculum vulgare* (2 mithqāls of root bark and 5 mithqāls of seed) + *Apium graveolens* (2 mithqāls of root bark and 5 mithqāls of seed) + *Pimpinella anisum* (5 mithqāls)	Boil the ingredients in a bowl of water until the water reduces by half. Strain and mix with 70 mithqāls of white sugar until it reaches a syrupy consistency. Dissolve a spoonful in seven spoonfuls of Araq^a^-e-Chicory (*Cichorium intybus*) every morning and take it.
Sharbat-e-bozouri har 2	*Foeniculum vulgare* (2 derhams of seed and 4 derhams of root) + *Cichorium intybus* (2 derhams of seed and 4 derhams of root) + *Cucumis sativus* (seed) (2 derhams) + *Cucumis melo* (seed) (2 derhams) + *Apium graveolens* (1 derham of seed and 1 derham of root) + *Pimpinella anisum* (1 derham) + *Capparis spinosa* (1 derham)	Soak the half-ground ingredients overnight, then boil and strain with half a cup of white sugar to thicken. Each half-ratl ingredient requires 4 derhams of white sugar.
Ma’al osool	*Foeniculum vulgare* (4 mithqāls of seed and 5 mithqāls of root bark) + *Apium graveolens* (seed) (4 mithqāls) + *Pimpinella anisum* (seed) (4 mithqāls) + *Capparis spinosa* (root bark) (5 mithqāls) + *Glycyrrhiza glabra* (3 mithqāls) + *Vitis vinifera* (without seed) (37 mithqāls) + *Ficus carica* (20 pieces) + *Cichorium intybus* (3 mithqāls) + *Cicer arietinum* (skinless) (10 mithqāls) + *Hordeum vulgare* (hulled) (7 mithqāls) + *Adiantum capillus-veneris* (3 mithqāls)	Those ingredients that need to be crushed are ground into a coarse powder and then boiled in 4 L of water until reduced to 2 L. After straining, it is ready for use.
Methroditus	*Crocus sativus* (10 derhams) + *Commiphora myrrha* (10 derhams) + Gharikon (10 derhams)^b^ + *Zingiber officinale* (10 derhams) + *Cinnamomum burmanni* (10 derhams) + *Astragalus gummifera* (10 derhams) + *Pistacia terebinthus* (10 derhams) + *Nardostachys jatamansi* (8 derhams) + *Sinapis alba* (8 derhams) + *Cymbopogon schoenanthus* (8 derhams) + *Commiphora gileadensis* or *Momordica balsamina* (8 derhams) + *Dolomiaea costus* or *Hellenia speciosa* (8 derhams) + *Lavandula stoechas* or *Nepeta menthoides* (8 derhams) + *Levisticum officinale* (8 derhams) + *Teucrium chamaedrys* (8 derhams) + *Piper longum* (8 derhams) + *Tragopogon* spp. or *Tragopogon graminifolius* (8 derhams) + Ratinaj (*Pinus* spp*.* or *Pinus gerardiana* Wall. ex D.Don or *Pinus halepensis* Mill) (8 derhams) + *Opopanax chironium* (8 derhams) + *Styrax officinale* or *Liquidambar orientalis* (8 derhams) + *Cinnamomum citriodorum* (8 derhams) + *Cinnamomum iners* or *Neolitsea cassia* (8 derhams) + *Senna tora* (20 derhams) + *Teucrium polium* or *Ajuga iva* (20 derhams) + *Colchicum autumnale* (20 derhams) + *Teucrium scordium* (20 derhams) + *Daucus carota* (20 derhams) + *Melilotus officinalis* (20 derhams) + *Gentiana lutea* (20 derhams) + Faldafiyoon^c^ (20 derhams) + *Commiphora mukul* (20 derhams) + *Ruta graveolens* (2 derhams) + *Ferula ammoniacum* (5 derhams) + *Nardostachys jatamansi* (5 derhams) + *Pistacia lentiscus* (5 derhams) + *Vachellia nilotica* (5 derhams) + *Petroselinum crispum* (5 derhams) + *Lagoecia cuminoides* (5 derhams) + *Papaver somniferum* (5 derhams) + *Foeniculum vulgare* (5 derhams) + *Rosa × damascena* (5 derhams) + *Origanum dictamnus* (5 derhams) + *Pimpinella anisum* (3 derhams) + *Acorus calamus* (3 derhams) + *Valeriana tuberosa* (3 derhams) + *Ferula persica* (3 derhams) + *Asarum europaeum* (3 derhams) + Sereh Saqanqir^d^ (4.5 derhams) + *Vachellia nilotica* (4.5 derhams of aqāqia) + Gharikon (4.5 derhams) + Sharab-e Reyhani^e^ (4 ratls) + Honey (5 ratls)	After soaking the resins mentioned in the formulation in the wine, mix all the ingredients with honey and store them. It can be used after 6 months. Its potency lasts up to 12 years. Take one nokhod per day.
Majoon-e-falaafeli	*Piper nigrum* (20 derhams of black pepper and 20 derhams of white pepper) + *Piper longum* (20 derhams) + *Commiphora gileadensis* or *Momordica balsamina* (10 derhams) + *Nardostachys jatamansi* (4 derhams) + Mineral antidote stone^f^ (4 derhams) + *Zingiber officinale* (1 derham) + *Apium graveolens* (1 derham) + *Cinnamomum iners* or *Neolitsea cassia* (1 derham) + *Levisticum officinale* (1 derham) + *Asarum europaeum* (1 derham) + *Inula helenium* (1 derham)	After the mixture has been crushed and sieved, it is mixed with three times the amount of honey. Take one derham per day.
Mask-albawl-e-Har	*Quercus ilex* (50 derhams) *+ Boswellia sacra* (50 derhams) *+ Prunus mahaleb* (5 derhams) + *Cyperus longus* (5 derhams) + *Commiphora myrrha* (5 derhams) + *Alpinia galanga* (5 derhams) + *Portulaca oleracea* (5 derhams) + *Elymus repens* (5 derhams) + *Inula helenium* (5 derhams)	All the ingredients are ground and mixed. It is recommended to take three derhams in the morning and three derhams in the evening every day. Taking this medicine with dried figs (*Ficus carica*) and seedless Maveez (*Vitis vinifera*) or half a derham of Suxenia Stone with two derhams of Triphala^g^ twice a day, along with Sharbat-e-bozouri, is recommended.
Safoof-e-zaranb	*Oxalis acetosella* (1 derham) + *Berberis vulgaris* (1 derham) + *Vitis vinifera* (1 derham) + *Coriandrum sativum* (1 derham) + *Rosa × damascena* (1 derham) + *Ceratonia siliqua* (1 derham) + *Quercus ilex* (1 derham) + *Bambusa bambos* (1 derham) + Rhus coriaria (2 derhams) + *Myrtus communis* (2 derhams) + *Punica granatum* (Flower) (4 derhams)	All the ingredients are ground, mixed, and sieved.
Dhiabitos 1 b y Hakim Sabit Ibn Qurra	*Myrtus communis* (2 derhams) + *Oxalis acetosella* (2 derhams) + *Vachellia nilotica* (1 derham) + Starch (1 derham) + *Plantago ovata* (1 derham)	The hulled seeds *of Myrtus communis* and *Oxalis acetosella* are crushed with *Vachellia nilotica* and starch, then mixed with *Plantago ovata glaze* to form tablets.
Dhiabitos 2	*Bambusa bambos* (10 derhams) + *Glycyrrhiza glabra* (10 derhams) + *Lactuca sativa* (2 derhams) + *Portulaca oleracea* (15 derhams) + *Rosa × damascena* (5 derhams) + *Coriandrum sativum* (5 derhams) + *Vachellia nilotica* (3 derhams) + *Santalum album* (3 derhams) + Armenian bole (3 derhams) + *Punica granatum* (flower) (3 derhams) + *Camphora officinarum* (0.5 derham)	Crush all ingredients, then mix with rosewater^h^ to form tablets. Take three derhams per day with sour pomegranate juice.
Dhiabitos 3	*Bambusa bambos* (10 derhams) + *Lactuca sativa* (15 derhams) + *Portulaca oleracea* (15 derhams) + *Coriandrum sativum* (5 derhams) + *Rosa × damascena* (2 derhams) + *Punica granatum* (flower) (2 derhams) + Armenian bole (5 derhams) + *Camphora officinarum* (0.5 derham)	Crush all ingredients and process them into tablets. Take two mithqāls per day with sour pomegranate juice.
Tabaashir	*Bambusa bambos* (10 derhams) + *Portulaca oleracea* (15 derhams) + *Lactuca sativa* (15 derhams) + *Coriandrum sativum* (5 derhams) + *Rosa × damascena* (5 derhams) + Armenian bole^i^ (5 derhams) + *Punica granatum* (flower) (2 derhams) + *Santalum album* (2 derhams) + *Vachellia nilotica* (2 derhams) + *Camphora officinarum* (0.5 derham)	Dry and roast *Coriandrum sativum* after soaking it in vinegar for 3 days. Combine all the powdered ingredients and form tablets. This formulation is effective for up to 6 months. Take one mithqal per day.
Hot Diabetes		
Sharbat-e-Onnāb	*Ziziphus jujuba* (1 ratl) + *Coriandrum sativum* (2 ūqiyyahs) + *Vicia lens* (2 ūqiyyahs) + *Cichorium intybus* (root) (2 ūqiyyahs)	Boil the ingredients in ten ratls of water until the volume is one-third. After straining, thicken with sugar to the final weight. The potency lasts for 2 months.
Sharbat-e-khashkhash	*Papaver somniferum*	One hundred ripe poppies are crushed with their seeds, or the husks are partially crushed separately, and the seeds are finely ground. Then, boil it with ten times the quantity of rainwater (spring rainwater) until it is reduced to a third, and with the same quantity, prepare the sugar to the right consistency. The amount of syrup is around twenty mithqāls, and its potency lasts up to 2 years.
Sharbat-e-Mufarreh	*Punica granatum* (juice of sweet and sour pomegranates, 0.5 pav each) + *Prunus domestica* (7 pavs) + *Tamarindus indica* (seed) (4 tolās) + Rangtareh juice (0.5 istār) + white rock sugar (0.5 istār) + Anbar-e-ashhab^j^ (ambergris) (0.5 māsheh) + *Oxalis acetosella* (5 māshehs) + *Coriandrum sativum* (peeled seed) (6 māshehs) + Mineral antidote stone (6 māshehs) + *Cucurbita pepo* (seed kernel) (6 māshehs) + *Cucumis sativus* (seed kernel) (6 māshehs) + *Cassia fistula* (seed kernel) (6 māshehs) + *Portulaca oleracea* (peeled seed) (6 māshehs) + *Lactuca sativa* (seed) (6 māshehs) + *Bambusa bambos* (6 māshehs) + *Echium amoenum* (flower) (4 māshehs) + *Vateria indica* (3 māshehs) + Silver leaf sheet (3 māshehs) + *Lepidium virginicum* or *Alyssum alyssoides* or *Matthiola incana* (3 māshehs) + Yashm-e Sabz (green jade) (3 māshehs) + marvārid nāsofteh^k^ (3 māshehs) + Red coral (3 māshehs)	Mix the sweet and sour pomegranate juice, *Prunus domestica*, *Tamarindus indica*, Rangtareh juice, and thicken it with white rock sugar. Melt the ambergris and add it to the mixture along with other herbal and mineral ingredients.
Javaresh-e-ameleh	*Phyllanthus emblica* (10 mithqāls) + *Berberis vulgaris* (5 mithqāls) + *Camphora officinarum* (0.5 mithqāl) + *Crocus sativus* (0.5 mithqāl) + *Rosa × damascena* (3 mithqāls) + *Bambusa bambos* (1 mithqāl) + Mineral antidote stone (1 mithqāl) + Gel-e-daghestan^l^ (1 mithqāl) + *Vateria indica* (1 mithqāl) + Armenian bole (1 mithqāl) + Yashm-e sabz (green jade) (1 mithqāl) + Abresham-e-mogharraz^m^ (1 mithqāl) + *Santalum album* (1 mithqāl) + *Tamarix gallica* (1 mithqāl) + *Coriandrum sativum* (3 mithqāls) + *Portulaca oleracea* (3 mithqāls)	Minerals are ground with rosewater on a porphyry stone (sumac stone), herbs are ground, and the resulting mixture is combined with *Cydonia oblonga* and *Malus domestica* concentrated extracts (50 mithqāls each). Take two derhams per day.
Mask-albawl	*Boswellia sacra* + *Quercus ilex* + *Cyperus longus* + *Alpinia galanga* + *Portulaca oleracea* + honey	Mix the ingredients in equal parts with honey.
Khamireye abrisham	Raw silk from the cocoon (50 mithqāls) + Rosewater (1 ratl) + Araq-e-Gāv-zabān (*Echium amoenum*) (1 ratl) + Araq-e-Bidmeshk (*Salix aegyptiaca*) (1 ratl) + Araq-e-Badranjbuyeh (*Melissa officinalis*) (1 ratl) + Araq-e-Darchini (*Cinnamomum burmanni*) (1 ratl) + Araq-e-Faranjmishk (*Ocimum basilicum* or *Ocimum* × *africanum*) (1 ratl) + *Phyllanthus emblica* (Dried fruits without seeds) (5 mithqāls) + Halileh kaboli (*Terminalia chebula*) (5 mithqāls) + *Citrus × limon* or *Citrus medica* (Fruit peel) (5 mithqāls) + *Berberis vulgaris* (without seeds) (5 mithqāls) + *Ocimum basilicum* or *Ocimum × africanum* (5 mithqāls) + *Melissa officinalis* (5 mithqāls) + *Echium amoenum* (flower, Leaf, Root bark, 5 mithqāls of each) + *Rosa* × *damascena* (flower) (5 mithqāls) + *Nymphaea alba* (5 mithqāls) + *Zingiber zerumbet* (5 mithqāls) + *Centaurea behen* (5 mithqāls) + *Limonium vulgare* (5 mithqāls) + *Doronicum pardalianches* (5 mithqāls) + *Elettaria cardamomum* (3 mithqāls) + *Amomum subulatum* (3 mithqāls) + *Santalum album* (3 mithqāls) + Rosewater (*Rosa* × *damascena*) (3 mithqāls) + *Aquilaria malaccensis* (3 mithqāls) + white sugar (1 mann) + Asal-e-mosffa (Clarified honey)^n^ (0.5 ratl) + Apple wine (*Malus domestica*) (0.5 ratl) + Sweet Beh wine (*Cydonia oblonga*) (0.5 ratl) + *Rheum ribes* wine (0.5 ratl) + *Citrus × limon* or *Citrus medica* (0.5 ratl) + *Echium amoenum* wine (0.5 ratl) + *Melissa officinalis* wine (0.5 ratl) + Anbar-e-ashhab (Ambergris) (1 mithqāl) + Torkish Musk^o^ (1 mithqāl) + *Crocus sativus* (1 mithqāl)	The raw silk is separated from the cocoon and soaked in rosewater and different araqs for 3 days. It is then boiled until it has reduced by half. Afterwards, the silk is thoroughly kneaded and pressed, and the araqs are separated and stored. The plant ingredients are ground and boiled with silk in two manns of water until it reduces to one-third. Filter it and add one mann of white sugar, clarified honey, and assorted wines along with the araqs. Over gentle heat, simmer until thick. Lastly, add ambergris, musk, and saffron, and store it in a bottle. Take six derhams per day.
Safoof dafe’e dhiabitos 1	*Ficus racemosa* + Armenian bole + *Punica granatum* (flower) + *Punica granatum* (seed) + *Mangifera indica* + *Phyllanthus emblica*	Crush all the ingredients. Mix two parts of *Ficus racemosa* with one part of each other ingredient, then mix with equal amounts of sugar.
Safoof dafe’e dhiabitos 2	*Vachellia nilotica* (6 māshehs) + *Astragalus gummifera* (6 māshehs) + Strach (6 māshehs) + *Cichorium intybus* (Seed) (6 māshehs) + *Cucurbita pepo* (Seed kernel) (6 māshehs) + *Citrullus lanatus* (Seed kernel) (6 māshehs) + *Cucumis melo* (Seed kernel) (6 māshehs) + *Lactuca sativa* (6 māshehs) + *Nymphaea alba* (6 māshehs) + *Althaea officinalis* (Seed) (6 māshehs) + *Rosa × damascena* (6 māshehs) + *Echium amoenum* (Flower) (6 māshehs) + *Oxalis acetosella* (Seed) (6 māshehs) + *Santalum album* (6 māshehs) + *Coriandrum sativum* (6 māshehs) + Hajar ul Yahood^p^ (6 māshehs) + Armenian bole (6 māshehs) + *Areca catechu* (6 māshehs) + *Orchis mascula* (2 Tolās)	Make a powder by crushing all ingredients and mixing half their weight with white sugar. Take five māshehs with fresh water per day.
Safoof dafe’e dhiabitos 3	*Tinospora cordifolia* + *Syzygium cumini* + *Withania somnifera* (ash) + Dried powder of seashell + *Glycyrrhiza glabra* + *Vachellia nilotica* + *Astragalus gummifera* + *Echium amoenum* + marvārid nāsofteh + *Rosa × damascena* + *Coriandrum sativum* + *Portulaca oleracea* + *Santalum album* + *Camphora officinarum* + *Punica granatum* (flower) + Armenian bole + *Papaver rhoeas* (seed) + Pinus spp. or *Pinus gerardiana* Wall. ex D.Don or *Pinus halepensis* Mill (seed kernel) + *Curculigo orchioides* + *Celosia argentea*	Mix all ingredients in equal parts, grind, sieve, and mix. Take 6 māshehs with *Cucurbita pepo* juice per day.
Safoof dafe’e dhiabitos 4	*Curculigo orchioides* (1 Tolā) + *Asparagus racemosus* (0.5 Tolā) + *Phyllanthus emblica* (0.5 Tolā) + *Pistacia lentiscus* (0.5 Tolā) + *Tribulus terrestris* (0.5 Tolā) + *Tinospora cordifolia* (0.5 Tolā) *+ Liquidambar orientalis* (0.5 Tolā) *+ Bambusa bambos* (0.5 Tolā) *+ Gentiana lutea* (0.5 Tolā) + Old well adobe^q^ (3 Tolās) + White sugar candy (3 Tolās)	All the ingredients are ground and mixed.
Safoof dafe’e dhiabitos 5	*Astragalus gummifera* + *Bambusa bambos* + Armenian bole	Mix all ingredients in equal parts, grind, sieve, and mix.
Safoof-e-Galoo	*Tinospora cordifolia* + Sugar	Take dried and thinly ground *Tinospora cordifolia* and combine it with the same amount of sugar; take two handfuls daily with lunch.
Safoof by Hakim Waris Ali Khan	*Bambusa bambos* (6 Māshehs) + *Pistacia lentiscus* (6 Māshehs) + *Astragalus gummifera* (6 Māshehs) + *Vachellia nilotica* (6 Māshehs of samq-e-arabi and 6 Māshehs of aqāqia) + Armenian bole (6 Māshehs) + *Tetraclinis articulata* (6 Māshehs) + *Punica granatum* (6 Māshehs of flowers and 6 Māshehs of sour seeds) + *Tamarix gallica* (6 Māshehs) + *Shorea robusta* (6 Māshehs) + *Dolomiaea costus* or *Hellenia speciosa* (6 Māshehs) + *Elettaria cardamomum* (6 Māshehs) + *Bombax ceiba* (6 Māshehs) + *Aquilaria malaccensis* (6 Māshehs) + *Berberis vulgaris* (without seeds) (6 Māshehs) + Javahr mohra^r^ (6 Māshehs) + *Plantago major* (9 Māshehs) + Roasted *Quercus infectoria* (9 Māshehs) + Roasted *Terminalia chebula* (9 Māshehs) + marvārid nāsofteh (9 Māshehs) + *Rhus coriaria* (7 Māshehs) + *Myrtus communis* (7 Māshehs) + Roasted *Cuminum cyminum* (7 Māshehs) + *Doronicum pardalianches* (7 Māshehs) + Roasted *Murraya paniculata* (7 Māshehs) + Roasted *Terminalia bellirica* (7 Māshehs) + Mineral antidote stone (khatayi) (5 Māshehs) + Anbar-e-ashhab (Ambergris) (5 Māshehs) + *Anemone coronaria* (5 Māshehs) + *Syzygium aromaticum* (1 Māsheh) + Zarvard^s^ (*Rosa × damascena*) (4 Māshehs) + Yashm-e Sabz (green jade) (4 Māshehs) + Bosad^t^ (*Corallium rubrum*) (3.5 Māshehs) + *Withania somnifera* (3.5 Māshehs) + Maveez (*Vitis vinifera*) (3.5 Māshehs) + Phitkari^u^ (3.5 Māshehs)	Crush all the ingredients and mix them into a powder. A daily dose of three to nine Māshehs is recommended. Furthermore, boil the crushed *Althaea officinalis* roots in water and four tolās of pure rosewater until it reduces by half. After straining, add almond kernel milk and drink.
Safoof-e-qali	Simab^v^ (1 Tolā) + Qali^w^ (1 Tolā) + *Elettaria cardamomum* (1 Tolā) + *Vateria indica* (1 Tolā) + marvārid (1 Tolā)	After crushing the tin and mercury, mix them with the other ingredients and soften with rose water. Take 1 māsheh in the morning and avoid sour and flatulent foods.
Qors-e-Golnar	*Punica granatum* (flower) (5 mithqāls) + *Vachellia nilotica* (3 mithqāls of samq-e-arabi and 4 derhams of aqāqia) + *Rosa × damascena* (6 mithqāls) + *Astragalus gummifera* (1.5 mithqāls)	Grind all the ingredients and form them into twenty-four tablets. Take four tablets each morning and evening with mucilage from *Plantago ovata*. If no results are achieved, repeat the process and increase the dosage to four tablets each time.
Qors-e-Kafour	*Bambusa bambos* + *Santalum album* + *Coriandrum sativum + Portulaca oleracea + Oxalis acetosella + Lactuca sativa + Cucumis sativus + Cucurbita pepo + Vachellia nilotica + Punica granatum* (flower) *+ Camphora officinarum*	Mix equal amounts of the peeled seeds of *Coriandrum sativum* and *Lactuca sativa*, the seed kernels of *Cucumis sativus* and *Cucurbita pepo*, along with the other ingredients based on the total volume of the desired final formulation. Then, grind the mixture in water to form tablets.
Nafs-ol dam	*Vateria indica* + *Vachellia nilotica* (aqāqia and samq-e-arabi) + *Bambusa bambos* + *Dracaena cinnabari* + *Astragalus gummifera* + *Punica granatum* (flower) + *Tragopogon* spp*.* or *Tragopogon graminifolius* + Armenian bole + Starch	Grind all ingredients in equal parts and mix with *Plantago major* and *Portulaca oleracea* juice to form tablets.
Boul-ol dam	*Cucumis sativus* (seed kernel) (4 derhams) + *Tectona grandis* (1 derham) + *Vicia sativa* (1 derham) + *Astragalus gummifera* (1 derham) + *Punica granatum* (flower) (1 derham) + *Dracaena cinnabari* (1 derham) + *Vachellia nilotica* (Samq-e-arabi) (1 derham)	Grind all ingredients and mix with the juice of *Plantago major* and *Portulaca oleracea* to form tablets.
Abzan nafe’e dhiabitos	*Hibiscus rosa-sinensis* (6 tolās) + *Nymphaea alba* (6 tolās) + *Gossypium herbaceum* (6 tolās) + Seda Golab (flower) (6 tolās) + *Rosa canina* L. (6 tolās) + Rangtareh (flower) (6 tolās) + *Cichorium intybus* (whole plant) (6 tolās) + *Lactuca sativa* (6 tolās of leaf and 6 tolās of seed) + *Vicia faba* (6 tolās) + *Althaea officinalis* (6 tolās of flower and 6 tolās of seed) + *Eleusine coracana* (6 tolās) + *Salix alba* (6 tolās) + *Malva sylvestris* (6 tolās) + *Cucumis sativus* (seed) (1 pav) + *Cucurbita pepo* (seed) (1 pav) + *Citrullus lanatus* (seed) (1 pav) + *Benincasa hispida* (seed) (1 pav)	The ingredients are boiled in water in a bath. The patient sits in or lies in the water after it becomes lukewarm. After the bath, the back and kidney areas are greased with an equal amount of flower oil (*Rosa × damascena)* and vinegar, and one or two cups of sour yogurt soda are given to drink.
Zemad-e-sandal	*Santalum album* + *Punica granatum* (flower) + *Vachellia nilotica* + Armenian bole + *Hordeum vulgare* + *Rosa × damascena* + *Coriandrum sativum*	Combine the ingredients in equal amounts as needed and gradually mix in rosewater (*Rosa × damascena*) and fresh *Coriandrum sativum* juice until a smooth, sticky poultice forms. Then apply it to the kidney area.
Zemad for dhiabitos	*Salix alba + Portulaca oleracea* (leaf) *+ Sempervivum tectorum + Cydonia oblonga + Hordeum vulgare + Vicia lens*	Mix all ingredients in equal amounts as needed, then gradually combine with vinegar, flower oil *(Rosa ×* damascena), and rosewater until a smooth, sticky poultice forms. Then apply it to the back and inner thighs.

^a^ Hydrodistillation of plants is known as “Araq” or “Hydrosol.”

^b^ Gharikon (*Laricifomes officinalis*) is a well-known medicinal mushroom in TPM.

^c^ It is a medicine consisting of noore (unrefined lime) (3 derham), Andarani salt (crystal rock salt) (1.5 derham), Shibb-e-Yamani^k^ (2derham), *Vachellia nilotica* (Aqāqia) (2.5 derham), *Commiphora myrrha* (3 derham), red and yellow zarnikh (a sulfur-like mineral that is red, yellow, orange or brown in color and appears transparent to semi-transparent, arsenic) (2.5 derham each), noshader (sal-ammoniac, ammonium chloride) (4 dānik). The ingredients are ground and mixed with vinegar to form a paste, which is then formed into tablets and dried.

^d^ Saqanqir is Roman. It is known as “Righ Mahi” in Persian. it is a reptile from the lizard family which lives in asia, Europe, and Africa. the back skin is usually pink or yellow with dark stripes, while the belly skin is white. Its well-known characteristics are found in the male caught in the spring before mating with the female. The belly was usually wiped clean of all entrails except for the back, filled with salt, and dried in the shade. The most effective parts of an animal are the lower back, the belly button and the tail base. Sereh Saqanqir means the Saqanqir belly button.

^e^ Grape wine with the scent of raw agarwood (*Aquilaria malaccensis*), cloves (*Syzygium aromaticum*), and other aromatic herbs.

^f^ Bezoar.

^g^ The term “Triphala” refers to medicines that contain *Phyllanthus emblica* L., *Terminalia chebula* Retz., and *Terminalia bellirica* (Gaertn.) Roxb. combined.

^h^ Since ancient times rosewater has been prepared from rose flowers (*Rosa × damascena*) by distillation.

^i^ A red earthy clay native to Armenia, valued for its medicinal properties and used as a pigment and base for gilding also.

^j^ It is a solid waxy substance found in sperm whale intestines (*Physeter macrocephalus*). Ambergris is used in Eastern cultures as a spice and for medicines; in the West, it is used to stabilize perfume scents (https://www.britannica.com/science/ambergris).

^k^ Refers to unrefined or natural pearls.

^l^ It is a type of clay that is usually white or milky in color, used to make medicine.

^m^ A silk cocoon finely shredded with scissors.

^n^ Wax-free and purified honey. Using a low flame, mix natural honey with water until some water evaporates and thickens. At the same time, foam is also removed.

^o^ Musk can be divided into four species. One species is called “Torki,” which is excreted by an animal resembling a Chinese deer, either through menstruation or hemorrhoids. It forms on rocks and is known for its exquisite fragrance, so intense that nosebleeds occur. This variety is characterized by a yellow color and firm texture, with elongated and thin pieces.

^p^ “Hajar ul Yahood” refers to a stone (*lapis judaicus*) larger than an olive, with lines running left and right on its surface. Some stones are round, while others are elongated. It becomes soft when placed into water. This stone is one of the oldest traditional medicinal remedies.

^q^ In the past, the adobe from old wells was removed, and after clearing the accumulated dirt, it was lightly crushed and submerged in water until it settled. The water was then drained, and the process was repeated five to seven times before the adobe was used.

^r^ The Serpent Stone.

^s^ “Zarvards” are small seeds found in rose flower centers (Achnese).

t Red coral.

^u^ Phitkari, Phitkari Safaid, Fitkari Safaid, Shibb-e-Yamani are names for Alum (zaj-e-sefid in Persian), also known as Aluminum Potassium Sulfate.

^v^ Mercury.

^w^ In Persian it is called “arzir,” “qali,” and in Hindi “rang.” There are two types: black and white. Black is lead and white is qali (tin, stannum).

**TABLE 5:** Conversion factor of each traditional weight unit to its modern equivalent.

Traditional weight unit	Modern equivalent weight
Mithqāl	4.547958 g
Māsheh	1.0042 g
Derham	3.183571 g
Tolā	12.0504 g
Pav	240 g
Ratl	409.31627 g
Ūqiyyah	34.10969 g
Nokhod	1.4865 g
Mann	3,000 g
Dānik	250 mg
Istār	20.88 g

### 3.3 Principles of formulating compound medicines in TPM

In TPM, all bodily functions are governed by the “ruling power,” which is regarded as the body’s primary healer and regulator. Its influence on the human organism is exerted through “the naturals,” a system of seven components. The first component is “the elements” (Arkan), with each of the four elements possessing distinct qualities, as previously described. The other components include the humors (Akhlat), temperaments (Mezaj), organs (A’za), spirits (Amah), faculties and forces (Qova), and functions (Afal). Together, these principles form the intellectual foundation of TPM theories, which serve as the basis for creating and formulating compound medicines. Like other Eastern medical traditions, TPM commonly employs compound medicines, particularly mixture herbal preparations. The formulation of such preparations is guided by two key determinants: the intrinsic characteristics of medicines and the complexity of the disease being treated. The intrinsic characteristics of medicines include six aspects. Firstly, regulatory effect: adjusting medicines’ potency by combining substances to achieve the desired effect. Secondly, corrective effects: counteracting dominant properties with adjuvants or supportive medicines. Thirdly, dispersing effects: improving the permeability of medicine and preventing dispersion by combining it with rapid penetration-enhancing organ-specific substances. Fourthly, detoxifying effect: neutralizing side effects or toxicity. Fifthly, pleasant effect: masking unpleasant odors or tastes with substances such as honey or saffron. Sixthly, protective effects on noble organs: reducing potency or strengthening sensitive organs with protective substances such as sugar or quince wine. In the case of disease complexity, there are three distinct aspects. Firstly, the disease consists of two humors: a single medicine cannot remove both humors alone. Secondly, the single medicine has two potencies: one weaker and one stronger in relation to the cause of the complex disease. It is necessary to combine it with a medicine that can attenuate both potencies. Chamomile, for example, has more resolve than its astringent property. So, if a strong astringent effect is required, it should be combined with an astringent. Thirdly, in complex diseases, one of the causes is stronger than the others. In this case, a combination of preparations that strengthen one of the potencies is required (Aghili Alavi Khorasani Shirazi, 2007; Paavilainen, 2009).

### 3.4 Mineral stones

In TPM, various minerals and stones are recognized for their therapeutic potential, particularly in the treatment of diabetes alongside botanical and animal-derived substances. Table 4 footnotes elaborate on several mineral sources, among which one notable example is the bezoar—a substance historically regarded as an antidote. This soft, layered stone resembles the structure of a pearl or an onion. While animal-derived bezoars are traditionally extracted from goats in India and from sheep and male goats in Iran, the reference here pertains specifically to a mineral-based bezoar. Known in Arabic as hajar al-sam, in Persian as Pādzahr kāni or FadZahar Madani, and in Indian traditional medicine as Zahar Mohra, this mineral antidote is attributed with medicinal properties and has been incorporated into TPM formulations. (Duffin, 2012). It is a mineral stone that occurs in different colors. It is found in regions such as China, Tibet, Kandahar, Kermanshah, Khorasan, Kerman, Turan, and Khulais, which are near Medina. The highest quality is “Khatayi.” Bezoar stones were highly regarded as alexipharmic agents in the 16th and 17th centuries (Duffin, 2013). In traditional medicine, bezoar is recognized for its properties that include clearing the heart, opening the body, cooling the liver, alleviating heat, and detoxifying both the liver and heart (Li et al., 2024). These effects appear to help restore balance when imbalanced humors arise during diabetes.

One of the most well-known healing clays is “Armenian bole,” which has been utilized for many purposes throughout history. In addition to its medicinal properties, this clay serves as a pigment, a base for oil painting, and in the gilding process (the application of gold leaf to intricately carved wooden items, often found on altarpieces and church altars). It is also employed in bookbinding (Gomes and Rautureau, 2021). Armenian bole is absorbent, astringent, detoxifying, antipyretic, and cooling (Hosseinkhani et al., 2025). In TPM, astringents are used to balance excessive moisture or fluidity in the body by promoting contractions and enhancing water retention in the renal system. Consequently, in cases of excess phlegmatic humor, the astringent properties of Armenian bole may help restore balance by reducing this excess.

Ambergris, a rare substance extracted from whale’s digestive tract, is traditionally valued for its invigorating effect. It is a dense, accumulating fecal mass sometimes found in both the Sperm Whale and the Pygmy Sperm Whale (Plön, 2022). This substance is classified as a cololite, which refers to a fecal mass that forms in the digestive tract. Ambergris can be divided into two subgroups: intestinalite (stored in the body cavity) and evisceralite (stored outside the carcass). Ambergris has been used for various purposes, including as medicine, a flavoring agent, an aphrodisiac, and a fixative in perfumes (Duffin, 2015). Ambergris has been used as a tonic for the brain, heart, and vital organs, and is also utilized in the treatment of neurological disorders (Kumar Singh et al., 2018). In traditional medicine, diabetes is considered a systemic disease that impacts multiple organs and systems. By strengthening the noble organs and addressing their weaknesses, ambergris may help the body manage the imbalances associated with diabetes more effectively.

Hajar ul Yahood is a fossilized stone traditionally recognized for its significant role in addressing urinary disorders, including urolithiasis. Mostly composed of calcium silicate, this mineral has been extensively used in Unani medicine to facilitate kidney stones’ dissolution, either as a powder or in combination with other therapeutic agents (Makbul et al., 2018). Considering that TPM emphasizes the role of the kidneys and their temperament in the context of diabetes, the use of Hajar ul Yahood may have been viewed as enhancing kidney function and supporting overall kidney health in individuals with diabetes.

Table 4 shows that noble metals such as silver and heavy metals such as tin, mercury, and arsenic are used as medicines. In TPM, arsenic compounds are called red and yellow zarnikh, including golden arsenic sulfide (As_2_S_3_, yellow in color) and Realgar (As_4_S_4_, red arsenic) (Carter et al., 2021). Historically, poisons and toxic substances were commonly used as medicines in various ancient medical traditions, including Traditional Chinese Medicine and TPM. These substances were regarded as potent remedies for serious ailments, believed to restore humoral balance and detoxify the body, thereby promoting overall health. Drawing from alchemical traditions, they were seen as transformative agents capable of purifying the body. Practitioners based their practices on empirical observations, acknowledging that certain toxic substances could produce beneficial effects. This demonstrates a complex understanding of health, where the distinction between poison and medicine was often unclear (Liu, 2021).

### 3.5 Medicinal plants in diabetes treatment

A total of 208 plant species from 81 botanical families have been identified as individual remedies or used in combination. Table 6 summarizes their traditional and common names, scientific classifications, botanical families, plant parts used, and temperaments as cited in TPM sources. The four most represented families are Fabaceae (14 species, 7%), Asteraceae and Apiaceae (13 species each, 6.5%), and Lamiaceae (12 species, 6%). The most utilized plant parts include seeds (31%), roots (22%), and fruits (17.5%). While all plants listed in Table 6 were recommended for managing both types of diabetes, either alone or in combination, Table 4 highlights certain preferred species that are more frequently cited in the dosages and formulations for both types of diabetes. These species include *Astragalus gummifera* Labill. (Moderate, slightly wet), *Bambusa bambos* (L.). Voss (Cold-dry), *Berberis vulgaris* L. (Warm-dry), *B. sacra* Flück. (Warm-dry), *Camphora officinarum* Boerh. ex Fabr. (Cold-dry), *Cichorium intybus* L. (Warm-dry), *Coriandrum sativum* L. (Cold-dry), *Crocus sativus* L. (Warm-dry), *Cucumis sativus* L. (Cold-wet), *Cyperus longus* L. (Warm-dry), *Glycyrrhiza glabra* L. (Warm-dry), *Hordeum vulgare* L. (Cold-dry), *Lactuca sativa* L. (Cold-dry), *Oxalis acetosella* L. (Cold-dry), *Papaver somniferum* L. (Whole plant: Cold-dry; Seeds: Cold-wet), *Portulaca oleracea* L. (Cold-dry), *Punica granatum* L. (Cold-dry), *Quercus ilex* L. (Warm-dry), *Rhus coriaria* L. (Cold-dry), *Rosa* × *damascena* Herrm. (Flower: Moderate, slightly Cold-dry; Rosewater: Moderate, slightly warm; Oil and Achnese: Warm-dry), *Santalum album* L. (Cold-dry), *Vachellia nilotica* subsp. tomentosa (Benth.) Kyal. & Boatwr. (Samq-e-arabi: Moderate, slightly dry; Aqāqia: Cold-dry), and *Vitis vinifera* L. (Warm-wet).

**TABLE 6:** Ethnopharmacological specifications of plant species used to treat diabetes in TPM.

No	Traditional name in TPM	Common name	Scientific name	Family	Used part	Temperament	References^a^
1.	Zaafaran	Saffron	*Crocus Sativus* L.	Iridaceae	Stigma, Flower	Warm-dry	6, 9, 10
2.	Morr-e-makki	Myrrh	*Commiphora myrrha* (T. Nees) Engl.	Burseraceae	Resin	Warm-dry	4, 9
3.	Zanjabil	Ginger	*Zingiber officinale* Roscoe.	Zingiberaceae	Rhizome	Warm-dry	4, 9
4.	Darchini, Qorfe	Cinnamon	*Cinnamomum burmanni* (Nees & T. Nees) Blume	Lauraceae	Bark	Warm-dry	6, 9, 10
5.	Katira	Tragacanth	*Astragalus gummifera* Labill.	Fabaceae	Gum	Moderate, slightly wet	5, 6, 7, 9, 10, 11
6.	Elk-ol-botm, Habbat-ol-khazra	Botm, Baneh	*Pistacia terebinthus* L.	Anacardiaceae	Fruit, Gum	Warm-dry	6, 9
7.	Sonbol (Sonbol-e-hendi, Nardin, Sonbol Ol-tib)	Spikenard	*Nardostachys jatamansi* (D. Don) DC.	Caprifoliaceae	Root	Warm-dry	6, 9
8.	Kondor	Frankincense	*Boswellia sacra* Flück.	Burseraceae	Resin	Warm-dry	6, 9
9.	Khardal-e-sefid	White mustard	*Sinapis alba* L.	Brassicaceae	Seed	Warm-dry	6, 9
10.	Ezkher	Adhkhur	*Cymbopogon schoenanthus* (L.) Spreng.	Poaceae	Root	Warm-dry	6, 9
11.	Ood-e-Balsaan	balm of gilead or Balsam apple	*Commiphora gileadensi* (L.) C. Chr or *Momordica balsamina* L.	Burseraceae Cucurbitaceae	Wood	Warm-dry	6, 9
12.	Ghost, kathah	Costus or cane-reed	*Dolomiaea costus* (Falc.) Kasana & A. K. Pandey or *Hellenia speciosa* (J. Koenig) S. R. Dutta	Asteraceae Zingiberaceae	Root, Oil	Warm-dry	6, 9
13.	Ostokhodus	Topped lavender	*Lavandula stoechas* L. or *Nepeta menthoides* Boiss. & Buhse	Lamiaceae	Flower	Warm-dry	6, 9
14.	Sisaliyos, Kashem	Lovagr	*Levisticum officinale* W. D. J. Koch	Apiaceae	Fruit	Warm-dry	4, 9
15.	Kamazarous	balout-ol-arz, camedrio	*Teucrium chamaedrys* L.	Lamiaceae	Root	Warm-dry	4, 9
16.	Dar-felfel	Long Pepper	*Piper longum* L.	Piperaceae	Fruit	Warm-dry	4, 9
17.	Javsheir	Hercules’ all-heal	Opopanax chironium (L.) W. D. J. Koch	Apiaceae	Exudate	Warm-dry	4, 9
18.	Miye-yābeseh or Miye-sāyeleh	storax	*Styrax officinale* L. or *Liquidambar orientalis* Mill.	Styracaceae Altingiaceae	Resin	Warm-dry	4, 9
19.	Sāzaj	Malabar Cinnamon	*Cinnamomum citriodorum* Thwaites	Lauraceae	Leaf	Warm-dry	6, 9
20.	Salikheh	Cassia or Darchini	*Cinnamomum iners* (Reinw. ex Nees & T. Nees) Blume or *Neolitsea cassia* (L.) Kosterm.	Lauraceae	Bark	Warm-dry	6, 9
21.	Jodeh	felty germander or Herb Ivy	*Teucrium polium* L or *Ajuga iva* (L.) Schreb.	Lamiaceae	Aerial parts	Warm-dry	6, 9
22.	Sourenjān	Autumn-crocus, Meadow-saffron	*Colchicum autumnale* L.	Colchicaceae	Flower	Warm-dry	6, 9
23.	Osqurdiyun	Water germander	*Teucrium scordium* L.	Lamiaceae	Bulb	Warm-dry	6, 9
24.	Doqu	Wild carrot	*Daucus carota* L.	Apiaceae	Tuber, seed		6, 9
25.	akleel-ul-mulk	Yellow sweet clover	*Melilotus officinalis* (L.) Lam.)	Fabaceae	Aerial parts	Warm-dry	6, 9
26.	Gentiana, Pekhan Bid, Yekhan Bid	Great yellow gentian	*Gentiana lutea* L.	Gentianaceae	Root	Warm-dry	6, 9, 10
27.	Moql	Guggulu	*Commiphora mukul* (Hook. ex-Stocks) Engl.	Burseraceae	Resin	Warm-dry	6, 9
28.	Sodāb	Rue	*Ruta graveolens* L.	Rutaceae	Seed, leaf	Warm-dry	6, 9
29.	Oshaq	Ammoniacum	*Ferula ammoniacum* (D. Don) Spalik, M. Panahi, Piwczyński & Puchałka	Apiaceae	Resin	Warm-dry	6, 9
30.	Mastaki	Mastic	*Pistacia lentiscus* L.	Anacardiaceae	Gum	Warm-dry	6, 9, 10
31.	Ommeqilān, Samq-e-arabi^b^	Gum-arabic tree	*Vachellia nilotica* subsp. tomentosa (Benth.) Kyal. & Boatwr.	Fabaceae	Resin, fruit, leaf	Samq-e-arabi: moderate, slightly dry aqāqia: cold-dry	4, 5, 7, 9, 11
32.	Fatrāsālion	Parsley	*Petroselinum crispum* subsp. crispum	Apiaceae	Seed	Warm-dry	6, 9
33.	Qerdemānā	Alaf-e-kaaji	*Lagoecia cuminoides* L.	Apiaceae	Fruit	Warm-dry	6, 9
34.	Afyoun, Koknar	Opium poppy	*Papaver somniferum* L.	Papaveraceae	Seed, seed coat	Whole plant: cold-dry seeds: cold-wet	6, 9, 11
35.	Rāziāneh	Common fennel	*Foeniculum vulgare* Mill.	Apiaceae	Root bark, Seed, root	Warm-dry	6, 9
36.	Gul-e-surkh	Damask rose	*Rosa × damascena* Herrm.	Rosaceae	Flower, oil, achnese	Flower: moderate, slightly cold-dry Rosewater: moderate, slightly warm Oil and achnese: warm-dry	5, 6, 7, 9, 10, 11
37.	Meshketarāmashi	Dittany of crete	*Origanum dictamnus* L.	Lamiaceae	Aerial parts	Warm-dry	6, 9
38.	Lehyatotteis	Sheng	*Tragopogon* spp. or *Tragopogon graminifolius* DC.	Asteraceae	Aerial parts	Warm-dry	6, 9, 11
39.	Anisoun	Anise	*Pimpinella anisum* L.	Apiaceae	Seed	Warm-dry	6, 9
40.	Vaj	Sweet flag	*Acorus calamus* L.	Acoraceae	Root	Warm-dry	6, 9
41.	Mov	—	*Valeriana tuberosa* L.	Caprifoliaceae	Root	Warm-dry	6, 9
42.	Fovah	Sonbol-e-jabali	*Valeriana italica* Lam.	Caprifoliaceae	Root	Warm-dry	6, 9
43.	Sakbineh, Sakbinaj	—	*Ferula persica* Willd.	Apiaceae	Gum	Warm-dry	6, 9
44.	Asarun	Asarabacca	*Asarum europaeum* L.	Aristolochiaceae	Rhizome	Warm-dry	6, 9
45.	Balout	Holly oak	*Quercus ilex* L.	Fagaceae	Husk of fruit, Fruit	Warm-dry	6, 9
46.	Habb-ol-mahlab	Perfumed cherry	*Prunus mahaleb* L.	Rosaceae	Seed	Warm-dry	6, 9
47.	Sood	Sweet cyperus	*Cyperus longus* L.	Cyperaceae	Root	Warm-dry	6, 9
48.	Kholanjān	Galangal	*Alpinia galanga* (L.) Will.	Zingiberaceae	Root	Warm-dry	6, 9
49.	Rāsan	Elecampane	*Inula helenium* L.	Asteraceae	Root	Warm-dry	6, 9
50.	Anjeir	Common fig	*Ficus carica* L.	Moraceae	Leaf, fruit	Warm-wet	3, 6
51.	Maveez^c^	Common grape vine	*Vitis vinifera* L.	Vitaceae	Fruit, seed	Warm-wet	6, 9
52.	Āqerqerhā	Akarkara	*Anacyclus pyrethrum* (L.) Lag.	Asteraceae	Root	Warm-dry	6, 9
53.	Kāsni, Hendabā	Chicory	*Cichorium intybus* L.	Asteraceae	Seed, whole plant	Warm-dry	6, 9
54.	Qantorioun-e-raqeiq (saqeir)	Centaury	*Centaurium erythraea* Rafn	Gentianaceae	Aerial parts	Warm-dry	6, 9
55.	Shokāyi	Scotch thistle	*Onopordon acanthium* L.	Asteraceae	Fruit, root	Warm-dry	6, 9
56.	Zarnab, Sorkhdār	English yew	*Taxus baccata* L.	Taxaceae	Aerial parts	Warm-dry	6, 9
57.	Fovvah-ol-Sabaqien	Common madder	*Rubia tinctorum* L.	Rubiaceae	Root	Warm-dry	6, 9
58.	Beed anjeer	Castor bean	*Ricinus communis* L.	Euphorbiaceae	Seed	Warm-dry	6, 9
59.	Senna Makki	Senna leaf	*Senna alexandrina* var. alexandrina	Fabaceae	Leaf	Warm-dry	6, 9
60.	Rivand khatai	Rhubarb	*Rheum palmatum* L.	Polygonaceae	Root	Warm-dry	6, 9
61.	Khiar chambar^d^, Amaltaas	Golden shower	*Cassia fistula* L.	Fabaceae	Seed kernel	Warm-wet	6, 9, 11
62.	Taranjabin	Camelthorn	*Alhagi maurorum* Medik	Fabaceae	Manna	Warm-wet	6, 9
63.	Lowz-ol-holv, Lowz-ol-morr	Almond	*Prunus amygdalus* Batsch	Rosaceae	Seed (oil)	Warm-wet	6, 9
64.	Bādrangbouyeh	Lemon balm	*Melissa officinalis* L.	Lamiaceae	Leaf	Warm-dry	6, 9, 10
65.	Nokhod	Chickpea	*Cicer arietinum* L.	Fabaceae	Seed	Warm-dry	6, 9
66.	Jadvār, Zarambad	Zedoaria	*Curcuma zedoaria* (Christm.) Roscoe	Zingiberaceae	Rhizome	Warm-dry	6, 9
67.	Satar farsi	Oregano	*Origanum vulgare* L.	Lamiaceae	Leaf	Warm-dry	6, 9
68.	Afsantin	Absinthe, Wormwood	*Artemisia absinthium* L.	Asteraceae	Aerial parts	Warm-dry	6, 9
69.	Dam-ol-akhaveyn, Khoun-e-siāvashān	Dragon blood tree	*Dracaena cinnabari* Balf. f.	Asparagaceae	Resin	Cold-dry	6, 9, 11
70.	Hel, Qaqeleh saghir, Khair buwa, Hil, Elaichi, Hamāmā	Cardamom	*Elettaria cardamomum* (L.) Maton	Zingiberaceae	Seed	Warm-dry	6, 9, 10
71.	Qaqeleh kebaar	Black cardamom, Bengal cardamom	*Amomum subulatum* Roxb.	Zingiberaceae	Seed	Warm-dry	6, 10
72.	Qennab, Shāhdāneh	Cannabis	*Cannabis sativa* L.	Cannabaceae	Seed	Warm-dry	6, 9
73.	Kākenaj	Chinese-lantern	*Alkekengi officinarum* Moench	Liliaceae	Fruit	Cold-dry	6, 9, 11
74.	Khosyosalab	Garden tulip	*Tulipa gesneriana* L.	Liliaceae	Root	Warm-wet	6, 10
75.	Khashkhāsh, Koknare-surkh	Corn poppy	*Papaver rhoeas* L.	Caryophyllaceae	Aerial parts, Seed	Cold-dry	6, 9
76.	Basfāyej	Common polypody	*Polypodium vulgare* L.	Polypodiaceae	Rhizome	Warm-dry	6, 9
77.	Darounaj aqrabi	Leopard bane	*Doronicum pardalianches* L.	Asteraceae	Root	Warm-dry	6, 9, 10
78.	Oshbeh-maqrebiyeh	Cat greenbrier	*Smilax glauca* Walter	Smilacaceae	Root	Warm-dry	6, 9
79.	Shaqāqol	Field eryngo	*Polygonatum orientale* Desf. or *Eryngium campestre* L.	Asparagaceae Apiaceae	Herb, root	Warm-wet	6, 9
80.	Bahman-e-sorkh	Bahman or Sea Lavender	*Limonium axillare* (Forssk.) Kuntze or *Limonium vulgare *Mill.	Plumbaginaceae	Root	Warm-dry	6, 9
81.	Bahman-e-sefid	Behman safed	*Centaurea behen* L.	Asteraceae	Root	Warm-dry	6, 9, 10
82.	Bahman-e-sorkh	Common sea-lavender	*Limonium vulgare* Mill.	Plumbaginaceae	Root	Warm-wet	6, 9, 10
83.	Jowz-e-bouya	Nutmeg	*Myristica fragrans* Houtt.	Myristicaceae	Seed	Warm-dry	6, 9
84.	Kazmazaj, Tarfā	French tamarisk	*Tamarix gallica* L.	Tamaricaceae	Aerial parts, Fruit	Warm-dry	6, 10
85.	Mourd, Aas	Myrtle	*Myrtus communis* L.	Myrtaceae	Leaf	Warm-dry	5, 6, 9, 10
86.	Sarv	Italian cypress	*Cupressus sempervirens* L.	Cupressaceae	Leaf, Fruit	Warm-dry	6, 10
87.	Banj, Bang-daneh	Henbane or White henbane	*Hyoscyamus niger* L. or *Hyoscyamus albus* L. or *Hyoscyamus Aureus* L.	Solanaceae	Fruit, leaf	Cold-dry	6, 10
88.	Holbeh	Fenugreek	*Trigonella foenum-graecum* L.	Fabaceae	Leaf, seed	Warm-dry	6, 10
89.	Bābounaj, Bābouneh	Chamomile	*Chamaemelum nobile* (L.) All. or *Matricaria chamomilla* L.	Asteraceae	Flower	Warm-dry	6, 10
90.	Qasabozzarireh	Chiretta	*Swertia chirayita* (Roxb.) H. Karst.	Gentianaceae	Aerial parts	Warm-dry	6, 10
91.	Shuniz, Siāhdāneh	Black cumin	*Nigella sativa* L.	Ranunculaceae	Seed	Warm-dry	6, 10
92.	Faranjmishk, Shāhesfaram	Common basil or Lemon basil	*Ocimum basilicum* L. or *Ocimum × africanum* Lour.	Lamiaceae	Seed, leaf, flowering branches	Warm-dry	6, 10
93.	Bāqela	Broad bean	*Vicia faba* L.	Fabaceae	Seed	Cold-wet	6, 9
94.	Chalghuz, Sanawber, Ratinaj ^e^	Chilghoza, Aleppo pine	*Pinus* spp. or *Pinus gerardiana* Wall. ex D. Don or *Pinus halepensis* Mill.	Pinaceae	Seed kernel, resin	Warm-dry	6, 10
95.	Konjed, Semsem	Sesame	*Sesamum indicum* L.	Pedaliaceae	Seed	Warm-wet	6, 10
96.	Hendevāneh, Tarbuz Betteikh-e-hendi	Watermelon	*Citrullus lanatus* (Thunb.) Matsum. & Nakai	Cucurbitaceae	Seed, seed kernel	Cold-wet	6, 9
97.	Betteikh, Kharbozeh	Muskmelon	*Cucumis melo* L.	Cucurbitaceae	Seed, seed kernel	Warm-wet	6, 10
98.	Bostan afrooz	Joseph’s coat or Ted amaranth	*Amaranthus tricolor* L. or *Amaranthus cruentus* L.	Amaranthaceae	Whole plant	Cold-dry	6, 10
99.	Khatmi	Marsh mallow	*Althaea officinalis* L.	Malvaceae	Flower, root, Seed	Cold-wet	6, 9, 10
100.	Aspqoul, Bazreqatounā	Psyllium	*Plantago ovata* Forssk.	Plantaginaceae	Seed	Cold-wet	5, 6, 7, 11
101.	Panj-angosht, Athlaq	Chaste tree	*Vitex agnus-castus* L.	Lamiaceae	Fruit	Warm-dry	6, 10
102.	Ehreiz, Kāfsheh, Qortom	Safflower	*Carthamus tinctorius* L.	Asteraceae	Flower	Warm-dry	6, 10
103.	Bondoq, Fandoq, Jolloz	European hazelnut	*Corylus avellana* L.	Betulaceae	Fruit	Warm-dry	6, 10
104.	Kāfour	Camphor tree	*Camphora officinarum* Boerh. ex Fabr.	Lauraceae	Exudate	Cold-dry	5, 6, 7, 10, 11
105.	Zireh sefid, Zireh sabz, karviā kammoon	Cumin or zeera	*Cuminum cyminum* L.	Apiaceae	Seed	Warm-dry	2, 6
106.	Tabāsheir, Bansalochan	Bamboo	*Bambusa bambos* (L.) Voss	Poaceae	Aerial parts (manna)	Cold-dry	5, 6, 7, 10, 11
107.	Sous, Shirin-bayān, Mahak	Liquorice	*Glycyrrhiza glabra* L.	Fabaceae	Root	Warm-dry	5, 6, 7, 11
108.	Kāhou, Khas	Lettuce	*Lactuca sativa* L.	Asteraceae	Seed, leaf	Cold-dry	5, 6, 7, 9, 11
109.	Khorfeh, Baqlat-ol-hamqā	Common purslane	*Portulaca oleracea* L.	Portulacaceae	Seed, leaf	Cold-dry	5, 6, 7, 9, 10, 11
110.	Geshneiz	Coriander	*Coriandrum sativum* L.	Apiaceae	Seed	Cold-dry	5, 6, 7, 9, 10, 11
111.	Sandal-e-sefid	White sandalwood	*Santalum album* L.	Santalaceae	Wood	Cold-dry	5, 6, 7, 9, 10, 11
112.	Sandal-e-sorkh	Red sandalwood	*Pterocarpus santalinus* L. f.	Fabaceae	Wood	Cold-dry	6, 9
113.	Anār, Golnār	Pomegranate	*Punica granatum* L.	Lythraceae	Seed, flowers, juice	Cold-dry	5, 6, 7, 9, 10, 11
114.	Hommāz, Torshak	Wood sorrel	*Oxalis acetosella* L.	Oxalidaceae	Leaf, seed	Cold-dry	5, 6, 7, 11
115.	Somāq	Sumac	*Rhus coriaria* L.	Anacardiaceae	Seed	Cold-dry	
116.	Khiar, Khiar badrang	Cucumber	*Cucumis sativus* L.	Cucurbitaceae	Seed, seed kernel	Cold-wet	6, 7, 11
117.	Kadou, Qar	Squash	*Cucurbita pepo* L.	Cucurbitaceae	Seed, seed kernel	Cold-dry	5, 6, 7, 11
118.	Felfel (Sefid/siyah)	Black pepper, White pepper^f^	*Piper nigrum* L.	Piperaceae	Fruit	Warm-dry	6, 9
119.	karafs-e-mamouli, karafs-e-bostāni	Celery	*Apium graveolens* L.	Apiaceae	Root, root bark, seed	Warm-dry	6, 9
120.	Zereshk	Common barberry	*Berberis vulgaris* L.	Berberidaceae	Root bark	Warm-dry	6, 10
121.	Kharnoub-e-shāmi	Carob-tree	*Ceratonia siliqua* L.	Fabaceae	Fruit	Cold-dry	6, 9
122.	Jo, Shaeir	Common barley	*Hordeum vulgare* L.	Poaceae	Seed	Cold-dry	6, 7, 11
123.	Bid	White willow	*Salix alba* L.	Salicaceae	Leaf	Cold-wet	6, 9, 10
124.	Bidmeshk	Musk willow	*Salix aegyptiaca* L.	Salicaceae	Flower	Cold-wet	6, 10
125.	Hai-ol-ālam, Abron	houseleek	*Sempervivum tectorum* L.	Crassulaceae	Herb	Warm-dry	6, 9, 11
126.	Safarjal, Beh	Quince	*Cydonia oblonga* Mill.	Rosaceae	Fruit, leaf	Warm-dry	6, 10
127.	Adas	Lentil	*Vicia lens* (L.) Coss. & Germ.	Fabaceae	Seed	Cold-dry	6, 11
128.	Gāv-zabān, Lesānossour	Persian borage	*Echium amoenum* Fisch. & C. A. Mey.	Boraginaceae	Flower, leaf, Root bark	Warm-wet	6, 9, 10, 11
129.	Ālou-bokhara	Plum	*Prunus domestica* L.	Rosaceae	Fruit	Cold-wet	6, 7, 10, 11
130.	Tamr-e-hendi	Tamarind	*Tamarindus indica* L.	Fabaceae	Seed	Cold-dry	6, 10, 11
131.	Limou, Utraj, Limoon	Lemon or Citron	*Citrus × limon* (L.) Osbeck or *Citrus medica* L.	Rutaceae	Fruit peel	Warm-dry	6, 10, 11
132.	Niloufar	White waterlily	*Nymphaea alba *L.	Nymphaeaceae	Flower	Cold-wet	6, 10
133.	Banafsaj, Banafsheh	Sweet violet	*Viola odorata* L.	Violaceae	Flower	Cold-wet	6, 10
134.	Nasrin, Gol-e-meshkin	Dog rose	*Rosa canina* L.	Rosaceae	Flower	Warm-dry	6, 7
135.	Āmeleh, Amlaj	Aamla	*Phyllanthus emblica* L.	Phyllanthaceae	Fruit	Cold-dry	6, 10
136.	Kewra, Kazi	kewda	*Pandanus odorifer* (Forssk.) Kuntze	Pandanaceae	Flower, stem	Warm-dry	6, 10
137.	Kahruba, Kahruba-i-shami	Kahruba, Dammar	*Vateria indica* L.	Dipterocarpaceae	Resinous exudate from stem, seed, root	Moderate, dry	6, 9, 10, 11
138.	Todari	Qadooma, Ghodumeh	*Lepidium virginicum* subsp. Virginicum or *Alyssum alyssoides* (L.) L. or *Matthiola incana* (L.) W. T. Aiton	Brassicaceae	Seed	Warm-wet	6, 9, 10, 11
139.	Arnabi	Clover, Rabbitfoot	*Trifolium arvense *L.	Fabaceae	Whole plant	Cold-dry	6, 10
140.	Tout	Mulberry	*Morus alba* L. *Morus nigra* L. Morus spp.	Moraceae	Fruit	Warm-wet	2, 6
141.	Nanā	Mint	*Mentha piperita* L.	Lamiaceae	Mint rob water	Warm-dry	2, 6
142.	Asarrāyi	Knotgrass	*Polygonum aviculare* L.	Polygonaceae	Aerial parts	Warm-dry	2, 6
143.	Sieb	Apple	*Malus domestica* (Suckow) Borkh	Rosaceae	Flower	Warm-wet	2, 6, 10
144.	Kanous	Common medlar	*Crataegus germanica* (L.) Kuntze	Rosaceae	Flower	Cold-dry	2, 6
145.	Sheng-e-tarei	Salsify	*Tragopogon porrifolius* L.	Asteraceae	Root	Cold-dry	2, 6
146.	Lāzan	Gum rock-rose, Ladanum	*Cistus ladanifer* L.	Cistaceae	Exudate	Warm-dry	2, 6
147.	Sebr	Aloe	*Aloe vera* (L.) Burm. f.	Asphodelaceae	Gel and juice extracted from leaf	Warm-dry	2, 6
148.	Turub	Radish	*Raphanus raphanistrum subsp. sativus* (L.) Domin	Brassicaceae	Root	Warm-wet	2, 6
149.	Habb-ol-bān	Drumstick tree	*Moringa oleifera* Lam. Or *Moringa* spp.	Moringaceae	Seed, oil	Warm-dry	2, 6
150.	Khandarous, Zorrat-e-makkeh, Solt, Jo-berahneh	Common wheat, bread wheat	*Triticum aestivum* subsp. Spelta (L.) Thell.	Poaceae	Fruit	Warm-dry	2, 6
151.	Alaf-e-limou	Lemon grass	*Cymbopogon citratus* (DC.) Stapf	Poaceae	Leaf	Warm-dry	2, 6
152.	Khormā, Rotab, Tamr	Date palm	*Phoenix dactylifera* L.	Arecaceae	Fruit	Warm-wet	2, 6
153.	Zeytoun	Common olive	*Olea europaea* L.	Oleaceae		Warm-dry	2, 6
154.	Arāk, Meswak,	Miswak, Mustard tree	*Salvadora persica* L.	Salvadoraceae	Branch	Warm-dry	2, 6
155.	Ribās, Rivās, Rivāj	Syrian rhubarb	*Rheum ribes* L.	Polygonaceae	Whole plant	Cold-dry	6, 10, 11
156.	Gular	Cluster fig	*Ficus racemosa* L.	Moraceae	Bark	Cold-dry	6, 10
157.	Anbeh	Mango	*Mangifera indica* L.	Anacardiaceae	Seed kernel	Warm-dry	6, 10
158.	Foufal, Sepāri	Areca	*Areca catechu* L.	Arecaceae	Flower	Cold-dry	6, 10
159.	Gilo khushk, Giloy, Guduchi, Sat gilo	Heart-leaved moonseed, Guduchi	*Tinospora cordifolia* (Willd.) Hook. f. & Thomson	Menispermaceae	Stem, leaf	-	6, 7, 10
160.	Jāmon	Jambolan	*Syzygium cumini* (L.) Skeels	Myrtaceae	Fruit	Cold-dry	6, 10
161.	Musali siyah	Golden eye-grass	*Curculigo orchioides* Gaertn	Hypoxidaceae	Rhizome	Warm-dry	6, 10
162.	Sarvali	Feather cockscomb	*Celosia argentea* L.	Amaranthaceae	Seed	Warm-dry	6, 10
163.	Satavar	Shatavari	*Asparagus racemosus* Willd	Asparagaceae	Root	Warm-dry	6, 10
164.	Lesān-ol-hamal, Bārhang	Common plantain	*Plantago major* L.	Plantaginaceae	Seed, leaf	Cold-dry	5, 6
165.	Sak, Sāj	Teak	*Tectona grandis* L. f.	Lamiaceae	Fruit	Cold-dry	6, 11
166.	Onnāb	Common jujube	*Ziziphus jujuba* Mill.	Rhamnaceae	Fruit	Moderate, slightly wet	6, 11
167. f	Khārkhāsak	Goathead	*Tribulus terrestris* L.	Zygophyllaceae	Fruit	Warm-dry	6, 10
168.	Salajet, Miye-sāyeleh	Oriental sweet-gum	*Liquidambar orientalis* Mill.	Altingiaceae	Resin	Warm-dry	6, 10
169.	Sandrous	Arartree	*Tetraclinis articulata* (Vahl) Mast.	Cupressaceae	Resin	Warm-dry	6, 10
170.	Ral sefaid, Qiqahar	Sal tree	*Shorea robusta* C. F. Gaertn.	*Dipte*rocarpacea*e*	Heartwood	Warm-dry	6, 10
171.	Mochras	Red silk cottontree	*Bombax ceiba* L.	Malvaceae	Exudate of bark (stem), flower, root	Cold-dry	6, 10
172.	Ood gharqi, Oud-e-hendi, Oud	Agarwood	*Aquilaria malaccensis* Lam.	Thymelaeaceae	Heartwood	Warm-dry	6, 10
173.	Mazuye sabz	Aleppoek	*Quercus infectoria* G. Olivier	Fagaceae	Gall	Cold-dry	6, 10
174.	Halileh zard, Halileh kaboli^g^	Myrobalan	*Terminalia chebula* Retz.	Combretaceae	Fruits (outer cover)	Cold-dry	6, 10
175.	Balileh	Beleric myrobalan, Bahera	*Terminalia bellirica* (Gaertn.) Roxb.	Combretaceae	Fruits (outer cover)	Cold-dry	6, 10
176.	Bilgari	Orange jasmine	*Murraya paniculata* (L.) Jack	Rutaceae	Fruit	Warm-dry	6, 10
177.	Shaqā'iq al-Na’mān	Common anemone	*Anemone coronaria* L.	Ranunculaceae	Flower, leaf	Warm-dry	6, 10
178.	Qaranfol	Clove	*Syzygium aromaticum* (L.) Merr. & L. M. Perry	Myrtaceae	Flower	Warm-dry	6, 10
179.	Farij, Marq	Couch grass	*Elymus repens* (L.) Gould	Poaceae	Aerial parts	Moderate, slightly cold	3, 6
180.	Fleawort	Branched plantain	*Plantago indica* L.	Plantaginaceae	Seed	Cold-wet	2, 6
181.	Nasha, Nafala	Common vetch	*Vicia sativa* L.	Fabaceae	Seed	Warm-dry	6, 10
182.	Neyb	Nutgrass	*Cyperus rotundus* L.	Cyperaceae	Rhizome	Warm-dry	6, 10
183.	Majlihah, hanzal, hendevāneh-aboujahl	Colocynth	*Citrullus colocynthis* (L.) Schrad.	Cucurbitaceae	Fruit	Warm-dry	6, 10
184.	Gul-e-gurhal, Angerae	Chinese hibiscus	*Hibiscus × rosa-sinensis* L.	Malvaceae	Flower	Moderate, slightly cold	6, 10
185.	Gol-e-panbeh baghi	Levant cotton	*Gossypium herbaceum L.*	Malvaceae	Flower	Warm-wet	6, 9
186.	khubazi	Common mallow	*Malva sylvestris* L.	Malvaceae	Seed	Cold-wet	6, 9
187.	Marjan, Eskandeh	Ashwagandha	*Withania somnifera* (L.) Dunal	Solanaceae	Root	Warm-dry	6, 10
188.	Badiyan-e-khatayi	Japanese star anise	*Illicium anisatum L.*	Schisandraceae	Fruit, seed	Warm-dry	6, 9
189.	Chay-e-khatayi	Tea plant	*Camellia sinensis* (L.) Kuntze	Theaceae	Leaf	Warm-dry	6, 9
190.	Zoronbād	Bitter ginger	*Zingiber zerumbet* (L.) Roscoe ex Sm.	Zingiberaceae	Root	Warm-dry	6, 9, 10
191.	Ashneh	Old Man’s Beard	*Usnea* spp. *Usnea barbata*	Usneaceae	Complete lichen	Moderate	6, 9
192.	Irsā	Florentine orris	*Iris Florentina* L.	Iridaceae	Root	Warm-dry	6, 9
193.	Bouzidān	Ashwagandha	*Withania somnífera* (L.) Dunal	Solanaceae	Root	Warm-dry	6, 9
194.	Choub-e-chini	China root	*Smilax china* L.	Smilacaceae	Root	Warm-wet	6, 9
195.	zoufā	Hyssop	*Dracocephalum officinale* (L.) Y. P. Chen & B. T. Drew	Lamiaceae	Aerial parts	Warm-dry	6, 10
196.	Kabar	Caperbush	*Capparis spinosa* L.	Capparaceae	Fruit, root bark	Warm-dry	6, 9, 10
197.	Paresiāvashān	Southern maidenhair fern	*Adiantum capillus-veneris* L.	Pteridaceae	Aerial parts	Moderate, slightly warm-dry	6, 9
198.	Sa lab-e-mesri	Early purple orchid	*Orchis mascula* (L.) L.	Orchidaceae	Root	Warm-wet	6, 9
199.	Kenab, Qennab	Finger millet	*Eleusine coracana* (L.) Gaertn.	Poaceae	Seed, leaf	Seed: warm-dry Leaf: cold-dry	6, 9
200.	Qelqelan	Sickle senna	*Senna tora* (L.) Roxb.	Fabaceae	Seed	Warm-wet	6, 9
201.	Pazoubeh, Paitha	wax gourd, winter melon	*Benincasa hispida* (Thunb.) Cogn.	Cucurbitaceae	Seed kernel, seed	-	6, 9
202.	Lak-e-Maghsool^h^	Lac, Shellac	*-*	-	-	Warm-dry	2
203.	Pare Khiar	—	—	—	—	—	9
204.	Gol Gost	—	—	—	—	—	9
205.	Sandal-e-zard	—	—	—	Wood	Cold-dry	9
206.	Pahalsah, Palsah	—	—	—	Root bark	Cold-dry	9
207.	Seda Golab	—	—	—	Flower	—	10
208.	Rangtareh, Kawnlah	—	—	—	Flower	Cold-wet	9

^a^ Correspond to the numbered books listed in Section 2.

^b^ This plant is referred to in TPM, books as “Samq-e-arabi,” and the extract of its fruit is called “Aqāqia.”

^c^ The dried black grapes are called “Maveez.” Two other parts of this plant are used in TPM: the green, unripe fruits and the shoots. Vinegar made from this plant is also widely used.

^d^ *Cassia fistula* L is called the Khiar chamber in TPM., nevertheless, the disorganized scaled flesh of its fruits is called “Felus.”

^e^ The resin of the Aleppo pine tree is called Ratinaj.

^f^ Black and white pepper come from the same plant, *Piper nigrum* L*.,* but are harvested and processed differently.

^g^ Halileh Siah, Halileh Zard and Halileh Kaboli originate from the same plant species. At the beginning, when the fruit is small, it is called “Halileh Siah.” When the fruit ripens and changes color, it is referred to as “Halileh Zard” when it turns yellow. Finally, when it is fully ripe and attains a brown hue, it is called “Halileh Kaboli.” The nomenclature for these myrobalans is thus inextricably linked to the specific growth at which they are harvested.

^h^ Purified materials obtained from the resinous secretion of the female insect *Kerria lacca* (Kerr) Lindinger (*Laccifer lacca* Kerr) which grows in South-East Asia on various tree species.

### 3.6 Proposing a framework for future studies

Considering the historical use of mixture herbal products by Persian practitioners for diabetes treatment, future research may focus on the individual constituents of these medicinal remedies. As the next step, we propose a separate review to systematically evaluate the phytochemical and pharmacological profiles of plants associated with the treatment of “hot” and “cold” diabetes, with a specific focus on their hypoglycemic effects. Plants used to treat “hot” diabetes, such as *Cucurbita pepo*, *Citrullus lanatus*, and *Melissa officinalis*, act as cooling agents and may possess anti-inflammatory properties that could contribute to the protection of pancreatic beta cells and the enhancement of insulin function. Future research should investigate these properties, particularly their ability to improve insulin sensitivity and mitigate oxidative stress, especially through mechanisms related to AMPK activation. Conversely, we recommend exploring plants associated with “cold” diabetes, such as *Zingiber officinale*, *Foeniculum vulgare*, and *Cinnamomum iners*, for their potential effectiveness in managing diabetes-related complications like nephropathy. Specifically, studies could investigate the antifibrotic properties of plants such as *Asparagus racemosus* in diabetic nephropathy models. By investigating the pharmacological and phytochemical profiles of these traditional remedies, we can gain a deeper understanding of their therapeutic potential and evaluate their integration into diabetes therapies. Additionally, exploring the synergistic interactions among these herbs in the context of mixed herbal products is essential. This approach will enhance our understanding of their collective efficacy and contribute to the development of informed practices in diabetes treatment with traditional herbal medicine.

## 4 Conclusion

This review highlights the historical perspective of TPM regarding diabetes, particularly its classification into “hot” and “cold” types. While TPM provides valuable insights into past therapeutic approaches, it is important to acknowledge that the concept of temperament may not align with contemporary evidence-based principles. This study identifies various dosage forms and ingredients used in traditional remedies, emphasizing the need for a deeper understanding of their potential pharmacological effects. In this study, approximately 208 plant species from 81 botanical families were identified as most traditional medicine remedies, which were presented as single or mixture herbal products for diabetes treatment. Each of these plants possesses at least one of several essential qualities believed by medieval healers to play an important role in the treatment of the disease. In addition, by correlating historical names of medicinal plants with contemporary equivalents, this review not only preserves cultural heritage, but also opens new avenues for exploring the therapeutic potential of these plants in modern herbal medicine. The concept of “hot and cold diabetes” likely represents the different stages of diabetes progression in modern literature. “Hot diabetes” aligns with early stages of Type 2 diabetes with symptoms such as frequent urination, intense thirst, and weight loss. In contrast, “cold diabetes” corresponds to advanced stages of Type 2 diabetes or possibly Type 1 diabetes with symptoms such as moderate thirst, neuropathy, and digestive problems. Future research should focus on the phytochemical and pharmacological profiles of plants associated with the treatment of “hot” and “cold” diabetes, emphasizing their hypoglycemic properties. Plants utilized for “hot” diabetes may exhibit anti-inflammatory effects that help protect pancreatic beta cells and enhance insulin function. Furthermore, it is important to investigate plants associated with “cold” diabetes for their potential effectiveness in managing diabetes-related complications, such as nephropathy.
